# Unexpected Phenotype of Mice Lacking Shcbp1, a Protein Induced during T Cell Proliferation

**DOI:** 10.1371/journal.pone.0105576

**Published:** 2014-08-25

**Authors:** Monica W. Buckley, Sanja Arandjelovic, Paul C. Trampont, Taeg S. Kim, Thomas J. Braciale, Kodi S. Ravichandran

**Affiliations:** 1 Department of Microbiology, Immunology, Cancer biology, University of Virginia, Charlottesville, Virginia, United States of America; 2 Carter Immunology Center, University of Virginia, Charlottesville, Virginia, United States of America; 3 Center for Cell Clearance, University of Virginia, Charlottesville, Virginia, United States of America; Baylor Institute for Immunology Research, United States of America

## Abstract

T cell development and activation are highly regulated processes, and their proper execution is important for a competent immune system. Shc SH2-domain binding protein-1 (Shcbp1) is an evolutionarily conserved protein that binds to the adaptor protein ShcA. Studies in Drosophila and in cell lines have strongly linked Shcbp1 to cell proliferation, embryonic development, growth factor signaling, and tumorigenesis. Here we show that Shcbp1 expression is strikingly upregulated during the β-selection checkpoint in thymocytes, and that its expression tightly correlates with proliferative stages of T cell development. To evaluate the role for Shcbp1 during thymic selection and T cell function *in vivo*, we generated mice with global and conditional deletion of *Shcbp1*. Surprisingly, the loss of Shcbp1 expression did not have an obvious effect during T cell development. However, in a mouse model of experimental autoimmune encephalomyelitis (EAE), which depends on CD4^+^ T cell function and mimics multiple features of the human disease multiple sclerosis, *Shcbp1* deficient mice had reduced disease severity and improved survival, and this effect was T cell intrinsic. These data suggest that despite the striking upregulation of Shcbp1 during T cell proliferation, loss of Shcbp1 does not directly affect T cell development, but regulates CD4^+^ T cell effector function *in vivo*.

## Introduction

Shcbp1 was first identified in a yeast-two hybrid screen designed to find new binding partners of the adapter protein ShcA, a critical regulator of T cell development [1], [2], [3]. Shcbp1 was initially named *murine Protein of Activated Lymphocytes* (mPAL), due to upregulation of its expression during T cell activation [1]. Shcbp1 is an evolutionarily conserved protein, with human SHCBP1 sharing 78% identity with mouse Shcbp1 and 23% identity with the Drosophila melanogaster homolog Nessun Dorma [4]. Nessun Dorma is an essential gene, as flies lacking Nessun Dorma exhibit partial lethality and defects in spermatogenesis, leading to infertility [4].

Recently, in unbiased screening assays, Shcbp1 has been identified in different contexts. These studies in mammalian cell lines and via genetic studies in Drosophila have implicated Shcbp1 in a diverse array of biological functions with links to proliferation and differentiation, including embryonic development, growth factor signaling, cytokinesis, spermatogenesis, tumorigenesis, and viral infections [1], [4], [5], [6], [7], [8], [9], [10]. In mammals, Shcbp1 was shown to be a regulator of proliferation induced by fibroblast growth factor signaling in neural progenitor cells [6]. In breast tumors of young women (who typically have more aggressive cancer and poorer prognosis), *SHCBP1* was shown to be upregulated in ductal carcinoma *in situ*, as well as in the invasive ductal carcinoma [7]. SHCBP1 was also found to be upregulated in human hepatocellular carcinoma (HCC) samples, and knockdown of SHCBP1 in HCC cell lines reduced cell proliferation and colony formation [9]. Microarray data have also suggested upregulation of *SHCBP1* in certain leukemia/lymphoma in both humans and mice [11], [12], [13], [14]. In peripheral T cell lymphomas, *SHCBP1* expression was higher in leukemic cells compared to both resting and activated peripheral T cells [11], [12], [13].

In gene expression databases, Shcbp1 expression also appears to correlate well with actively proliferating cells of the immune system, including developing thymocytes [13], [15]. Proliferation is precisely regulated during T cell development, as thymocytes undergo stages of active proliferation followed by temporary withdrawal from the cell cycle [16]. Developmentally, thymocytes undergo 4–6 cycles of proliferation after proceeding through the first developmental checkpoint, termed β-selection; β-selection ensures the productive rearrangement of the β-chain of the T cell receptor (TCR) [16], [17], [18], [19]. Proliferation during β-selection requires the preTCR and Notch-mediated signaling, as well as co-operation with other receptors including CXCR4 and IL7-R [20], [21], [22], [23], [24]. Recent studies have also shown that proliferation is absolutely required for differentiation during T cell development [19]. From a human health perspective, the proliferation that occurs during development is of interest since T cell leukemia and lymphoma often arise in the thymus during proliferative developmental stages [25], [26], [27], [28]. In fact, the preTCR is required for leukemic transformation in mice and most human T cell acute lymphoblastic leukemia express the preTCR [27], [29]. Thus, the correlation between Shcbp1 and proliferation is potentially relevant for these lymphomas.

Shcbp1 binds ShcA, an adaptor protein that functions as a critical regulator of T cell development [1], [2], [3]. ShcA is phosphorylated downstream of the preTCR as well as the chemokine receptor CXCR4 during β-selection, and relays signals essential for development [3], [22], [30]. In fact, ShcA is required for up to 70% of Erk activation in DN thymocytes [30]. In the absence of ShcA, there is a block in progression through the β-selection checkpoint, with impaired differentiation and reduced numbers of total thymocytes [2], [30].

While Shcbp1 has been linked to proliferation in different *in vitro* settings and in Drosophila, the *in vivo* function of Shcbp1 in mammals has not been elucidated. Given the expression of Shcbp1 in the thymus and the requirement of ShcA in T cell development, we hypothesized that Shcbp1 may be involved in the proliferation that occurs during T cell development or activation. After developing global and conditional *Shcbp1* deficient mice, we observed that, while Shcbp1 is induced in highly proliferative subsets during T cell development and is upregulated during β-selection, it is dispensable for T cell development *in vivo*. In a CD4^+^ T cell-driven EAE model, we find that Shcbp1 is upregulated in the spinal cords isolated from diseased mice, where it associates with inflammatory lesions, and Shcbp1 expression contributes to disease severity. Therefore, Shcbp1 regulates CD4^+^ T cell effector function in EAE without interfering with development or the proliferative state of activated T cells.

## Results

### Expression of Shcbp1 during T cell development tightly correlates with proliferative stages

**Figure 1:**
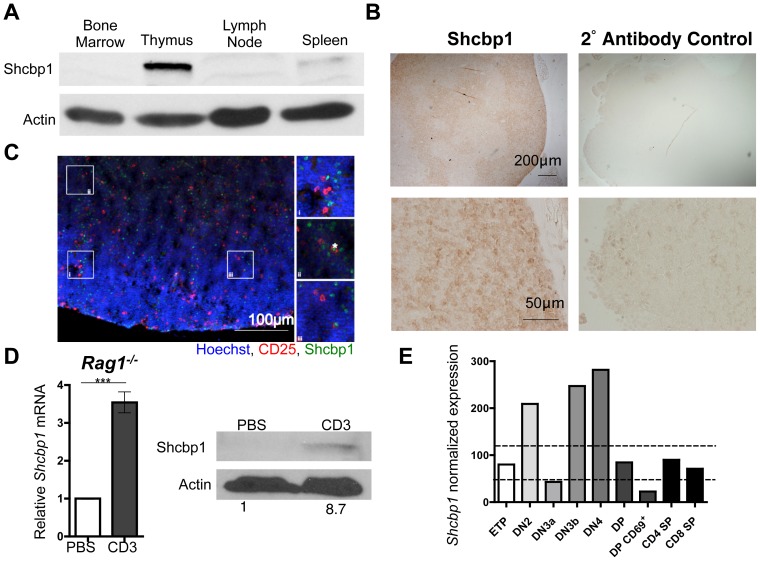
Expression of Shcbp1 during T cell development tightly correlates with proliferative stages. (A) Immunoblotting of Shcbp1 in the indicated tissues of a naïve C57BL/6J mouse (representative of n = 3 mice). (B) Left, immunohistochemistry of Shcbp1 in paraffin embedded thymic tissue from the C57BL/6J mouse (representative of n = 4 mice). Right, secondary antibody control staining. (C) Immunofluorescence of Shcbp1 and CD25 in frozen thymic tissue from the C57BL/6J mouse (representative of n = 2 mice). (D) Left, *Shcbp1* mRNA expression in thymocytes from *Rag1^−/−^* mice injected with either PBS or 100 µg of anti-CD3 antibody for 24 hours (n = 6 mice of each treatment, p<0.0001). Right, representative immunoblot of Shcbp1 with quantification. (E) *Shcbp1* mRNA expression analyzed by microarray in electronically sorted thymic subsets (data curated from the Immgen Database). Normalized expression value lower than 47 represents a gene that has a ≥95% probability of being a silent gene, while a normalized value of greater than 120 represents a gene that has a ≥95% probability of having true expression.

To determine the expression of Shcbp1 in different immune compartments, we analyzed lysates from the bone marrow, thymus, lymph node, and spleen of naïve C57BL/6J mice by immunoblotting. Expression of Shcbp1 was highest in the thymus compared to the other immune tissues (Figure 1A). We also examined the expression of Shcbp1 in the native architecture of the thymus, via immunohistochemistry and immunofluorescence analysis of thymic sections. Shcbp1 expression was most evident in the thymic outer cortex, which contains the CD4^−^CD8^−^ double negative (DN) thymocytes, as well as the CD4^+^CD8^+^ double positive (DP) thymocytes (Figure 1B) [16], [31]. When we co-stained for CD25, a cell surface marker expressed on DN2 and DN3 thymocytes, most of the CD25^+^ thymocytes did not express Shcbp1. Since most CD25^+^ thymocytes are non-proliferating DN3a thymocytes, this suggests that Shcbp1 is likely not expressed in the non-cycling or slowly cycling DN3a thymocytes (Figure 1C) [32]. However, CD25 is also expressed on the highly proliferative DN3b and DN2 thymocytes, and a subset of these CD25^+^ thymocytes expressed Shcbp1 (Figure 1C). These data suggested that Shcbp1 is expressed in the thymus within the actively proliferating thymic populations.

The increased expression of Shcbp1 in proliferating thymic subsets suggested that Shcbp1 might be up-regulated by preTCR signaling. To test this *in vivo*, we injected the antibody specific for CD3 (anti-CD3) into *Rag1^−/−^* mice (41). Thymocytes from *Rag1* deficient mice are arrested at the DN3 stage of T cell development, but express low levels of CD3, and crosslinking CD3 is sufficient to induce differentiation of the arrested DN3 thymocytes [33]. Shcbp1 was upregulated in thymocytes from anti-CD3 injected *Rag1^−/−^* mice, compared to the PBS injected *Rag1^−/−^* mice, as determined by RT-PCR and immunoblotting for Shcbp1 (Figure 1D). This suggested that Shcbp1 is induced after preTCR signaling in DN3 thymocytes.

We also examined the expression profile of *Shcbp1* in the different thymic subsets using data from the publicly available Immunological Genome Project Database (www.immgen.org) [15]. Again, *Shcbp1* expression highly correlated with thymocyte subsets known to be proliferative. Within the DN compartment, *Shcbp1* expression was increased in the highly proliferative DN2, DN3b, and DN4 compartments, but was likely not expressed in the non-cycling or slowly cycling DN3a compartment (Figure 1E). Previous studies have shown that β-selection represents a major transcriptional shift during thymocyte development, with 48% of the genes upregulated being related to proliferation [32]. We noted about a 6-fold increase in *Shcbp1* between the DN3a and DN3b compartment (Figure 1E). With respect to later thymic developmental stages, *Shcbp1* expression was low in the noncycling DP and SP compartments (Figure 1E). TCR signaling is also required in another thymic development checkpoint, namely positive selection at the DP stage. However, unlike β-selection, positive selection occurs in the absence of extensive proliferation [32]. Interestingly, there was no upregulation of *Shcbp1* in the transition from small DP to the positively-selected CD69^+^ DP thymocytes (Figure 1E) [15]. Collectively, these data suggest that *Shcbp1* expression in the thymus tightly correlates with the proliferative state of thymocytes.

### Shcbp1 expression is regulated by ShcA signaling in the thymus

**Figure 2:**
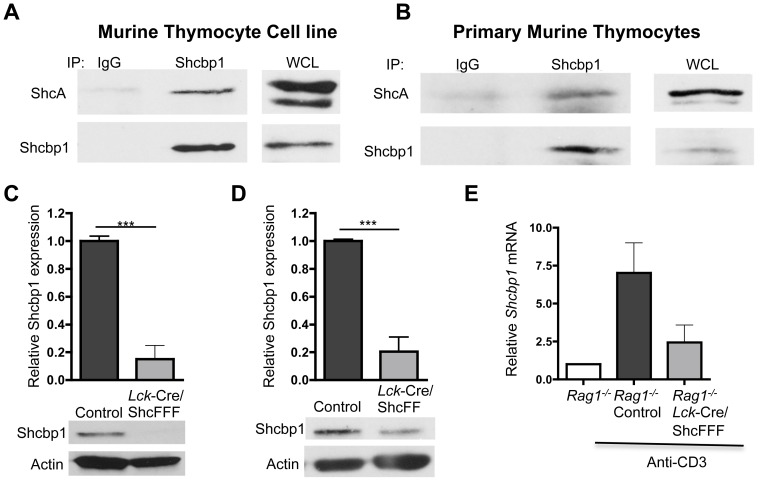
Shcbp1 expression is regulated downstream of ShcA signaling in the thymus. (A) Immunoprecipitation of Shcbp1 and immunoblotting for ShcA and Shcbp1 in the murine thymocyte cell line SCB29 (representative of n = 3 experiments). (B) Immunoprecipitation of Shcbp1 and immunoblotting for ShcA and Shcbp1 in primary murine thymocytes (representative of n = 2 experiments). (C) Shcbp1 expression in thymocytes expressing the ShcFFF transgene analyzed by immunoblotting in total thymocytes with quantification (n = 9 control and n = 5 *Lck*-Cre^+^/ShcFFF, p<0.0001). (D) Immunoblotting of Shcbp1 expression in thymocytes expressing the ShcFFF transgene, along with quantification (n = 4 control, n = 3 *Lck*-Cre^+^/ShcFF, p = 0.0003). (E) *Shcbp1* mRNA expression in thymocytes from *Rag1^−/−^* or *Lck*-Cre^+^/ShcFFF *Rag1^−/−^* mice injected with either PBS or 100 µg of anti-CD3 for 24 hours (n = 3 mice of each treatment, p = 0.07).

Shcbp1 was initially identified as a binding partner of ShcA, an adapter protein that relays signals downstream of many receptors including the TCR and the preTCR [1], [2]. Although ShcA binds to Shcbp1 in activated T cells [1], whether this interaction also occurs in the thymus was not known. Using the preTCR^+^ SCB29 murine thymocyte cell line and primary murine thymocytes, we found that ShcA binds to Shcbp1 in thymocytes (Figure 2A and 2B).

We next tested whether signaling via ShcA was required for Shcbp1 upregulation. We used transgenic mice with thymic expression of dominant negative forms of ShcA that cannot be phosphorylated on critical tyrosine residues (denoted ShcF_239_F_240_F_317_ (ShcFFF) and ShcF_239_F_240_ (ShcFF)). Thymocytes expressing the ShcFFF or ShcFF transgene have a block in T cell development at the DN3 stage of development [2], [30] along with a proliferative defect. We found that Shcbp1 expression was significantly reduced in thymocytes expressing the mutant ShcA transgenes compared to control thymocytes (Figure 2C and 2D). DNA microarray analysis of DN4 thymocytes expressing ShcFFF also showed that Shcbp1 expression was reduced 16-fold compared to control DN4 thymocytes. To test whether ShcA was required for Shcbp1 expression in the thymus, we crossed *Lck*-Cre/ShcFFF transgenic mice to *Rag1^−/−^* mice. In the *in vivo* model of anti-CD3-induced preTCR signaling, Shcbp1 was not upregulated to the same extent in *Rag1* deficient thymocytes expressing the ShcFFF transgene (Figure 2E). These data suggest that preTCR-induced upregulation of Shcbp1 requires optimal ShcA-mediated signaling.

### Generation of mice with conditional and global deletion of Shcbp1

**Figure 3:**
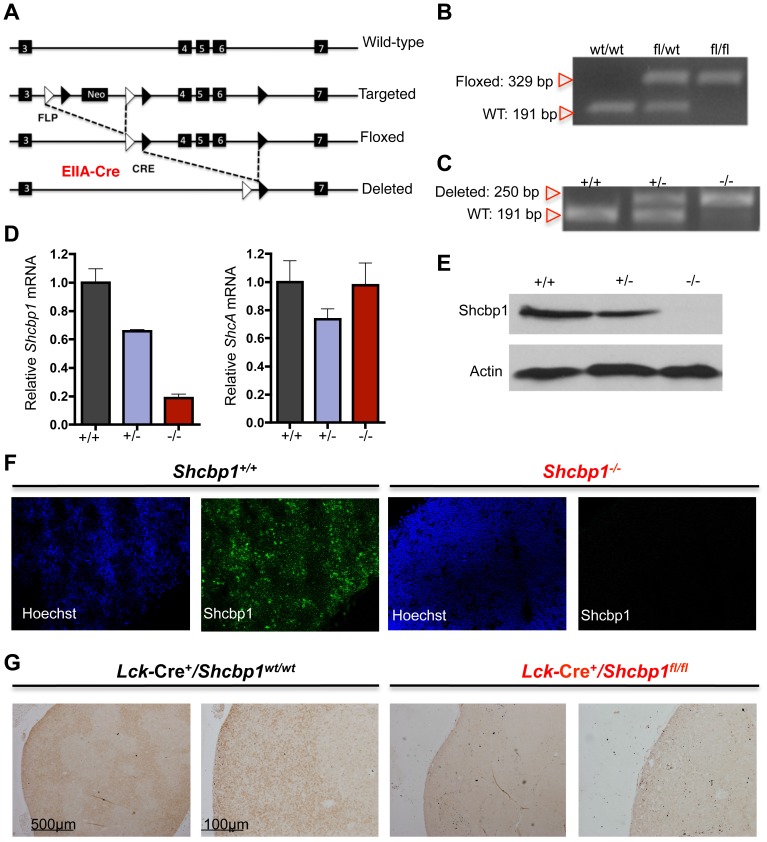
Generation of mice with conditional and global deletion of *Shcbp1*. (A) Schematic detailing the generation of mice carrying global deletion of *Shcbp1* and the strategy for conditional deletion of *Shcbp1* exons 4–6 and the neomycin targeting cassette. (B) Identification of mice with a floxed or WT *Shcbp1* loci (*Shcbp1* ^wt/wt^, *Shcbp1* ^fl/wt^, and *Shcbp1* ^fl/fl^) assessed by genotyping PCR in tail DNA. (C) Genotyping PCR for *Shcbp1* WT and *Shcbp1* deleted loci in tail DNA from mice with the indicated genotypes. (D) Left, *Shcbp1* mRNA levels in thymocytes isolated from *Shcbp1^+/+^*, *Shcbp1^+/−^*, and *Shcbp1^−/−^* mice normalized to *HPRT* and to control *Shcbp1^+/+^* mice (n = 2–5 mice per genotype). Right, *ShcA* mRNA levels in thymocytes isolated from mice of the indicated genotype normalized to *HPRT* and to control *Shcbp1^+/+^* mice (n = 2–5 mice per genotype). (E) Immunoblotting of Shcbp1 in total thymocytes from mice with the indicated genotypes (representative of n = 3 experiments). (F) Immunofluorescence of thymi from wild-type and *Shcbp1^−/−^* mice with staining for Hoechst and Shcbp1 (representative of n = 2 experiments). (G)Immunohistochemistry of Shcbp1 in thymic tissue from wild type and *Shcbp1*-deficient mice (representative of n = 3 experiments).

The striking expression of Shcbp1 within the proliferating populations of thymocytes and its upregulation in response to pre-TCR signaling suggested that Shcbp1 likely played a role in regulation of T cell development. To test this *in vivo*, we generated *Shcbp1* deficient mice. Since Shcbp1 is highly expressed in the embryo, and previous studies in Drosophila demonstrated that loss of the Shcbp1 homolog caused partial lethality [4], we chose to use the conditional knockout approach. We obtained embryonic stem cells with exons 4–6 of the *Shcbp1* locus flanked by *loxP* sites to generate the *Shcbp1* floxed mouse (Figure 3A, 3B) [34]. Cre-mediated deletion of these exons is designed to cause frame-shift mutations and the generation of multiple STOP codons, leading to ablation of protein expression. We crossed the *Shcbp1^fl/fl^* mouse line with the ubiquitously Cre expressing *EIIA*-Cre line to generate *Shcbp1^−/−^* mice [35] (Figure 3A, 3C). Surprisingly, *Shcbp1^−/−^* mice were viable and born in normal ratios (Figure S1). One possible reason for the lack of an obvious phenotype is the incomplete deletion of the *Shcbp1* locus or continued protein expression. We addressed this via several approaches. Shcbp1 expression by both mRNA and protein analysis was reduced in a dose dependent manner in the thymus of *Shcbp1^+/−^ and Shcbp1^−/−^* mice compared to *Shcbp1^+/+^* animals (Figure 3D, 3E). It is noteworthy that in the *Shcbp1^−/−^* mice, *ShcA* expression was unchanged (Figure 3D) [2], [30], [36], [37]. Loss of Shcbp1 expression was also confirmed by immunofluorescence (Figure 3F). Deletion of exons 4-6 of *Shcbp1* did not lead to a detectable truncated protein in thymocytes, as immunoblotting with antibodies to the N or C-terminus of Shcbp1 did not identify a band that might represent a truncated protein (Figure S2D). From these data, we concluded that we have successfully generated mice with deletion of Shcbp1 and complete ablation of Shcbp1 expression.

### Loss of Shcbp1 does not lead to an obvious impairment in T cell development

**Figure 4:**
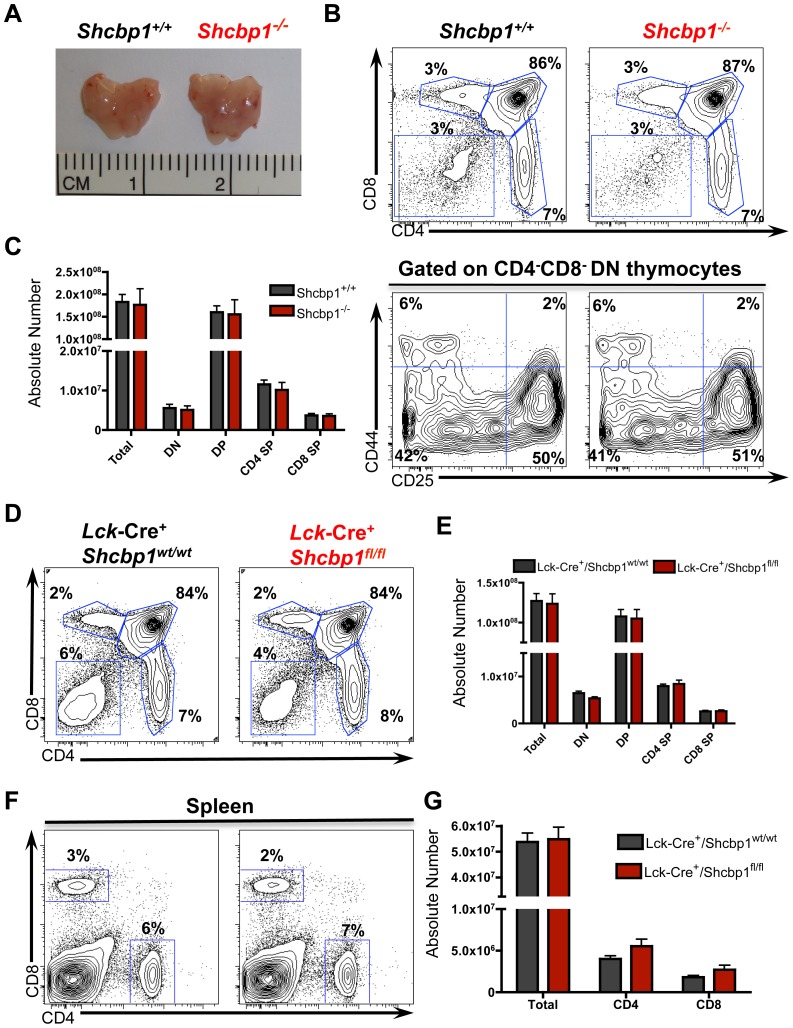
Loss of Shcbp1 does not impair T cell development. (A) Representative picture of the thymus from *Shcbp1^+/+^* and *Shcbp1^−/−^* mice. (B) Flow cytometry of thymi isolated from 4-to-6 week old *Shcbp1^+/+^* and *Shcbp1^−/−^* mice. Top panel is the surface marker expression of CD4 and CD8. Bottom panel shows surface marker expression of CD44 and CD25 gated on DN thymocytes (CD4^−^ CD8^−^ B220^−^ Gr1^−^ Ter119^−^ CD11b^−^ CD11c^−^). Data presented are representative of n = 4–6 mice per genotype of age-matched littermate controls. (C) Total cellularity and absolute number of thymic subsets in 4-to 6-week-old *Shcbp1^+/+^* and *Shcbp1^−/−^* mice (n = 4–6 mice of each genotype with age-matched littermate controls). (D) Flow cytometry of thymi isolated from *Lck*-Cre^+^/*Shcbp1^wt/wt^* and *Lck*-Cre^+^/*Shcbp1^fl/fl^* with analysis of cell surface markers CD4 and CD8 (representative of n = 11 mice per genotype of age-matched littermate controls). (E) Total cellularity and absolute number of thymic subsets in 4-to 6-week-old *Lck*-Cre^+^/*Shcbp1^wt/wt^* and *Lck*-Cre^+^/*Shcbp1^fl/fl^* mice (n = 11 mice of each genotype with age-matched littermate controls). (F) Flow cytometry for cell surface markers CD4 and CD8 in spleens isolated from 4-to-6-week old *Lck*-Cre^+^/*Shcbp1^wt/wt^* and *Lck*-Cre^+^/*Shcbp1^fl/fl^* mice (n = 11 mice of each genotype with age-matched littermate controls). (G) Absolute numbers of splenic CD4^+^ and CD8^+^ T cells as well as total splenocytes from *Lck*-Cre^+^/*Shcbp1^wt/wt^* and *Lck*-Cre^+^/*Shcbp1^fl/fl^* mice (n = 11 mice of each genotype).

Given the high Shcbp1 expression in proliferating thymocytes and its upregulation by preTCR signaling, we hypothesized that Shcbp1 would be required during thymocyte development. Much to our surprise, mice with a global deletion of Shcbp1 contained normal thymic appearance, cellularity and normal ratios of DN, DP, as well as CD4 and CD8 SP thymocytes (Figure 4A, 4B upper panel, 4C). In further analysis of the DN compartment, the fraction and absolute number of DN1, DN2, DN3, and DN4 subsets were largely unchanged (Figure 4B, lower panel). Additionally, TCRβ, CD3, and TCRγδ expression on thymocytes was comparable between *Shcbp1^+/+^* and *Shcbp1^−/^* ^−^ mice (Figure S3A, S3B). Consistent with the normal thymocyte composition, the overall structure and organization of the thymus appeared normal, as shown by the H&E staining of thymic cortex and medulla (Figure S3D).

To rule out potential compensatory effects in the global knockout mice and to directly test whether loss of Shcbp1 in developing thymocytes causes an effect, we crossed the *Shcbp1^fl/fl^* mice to either the *Lck*-Cre or the *Rag*-Cre transgenic mouse lines [38], [39]. The *Lck*-Cre transgenic mouse expresses Cre under the *lck*-proximal promoter from the DN1/DN2 stages of thymocyte development [38]. We first confirmed deletion of Shcbp1 in thymocytes from these mouse lines (Figure 3G, S2A-E, S4B-C). The *Lck*-Cre^+^/*Shcbp1^fl/fl^* and *Rag*-Cre^+^/*Shcbp1^fl/fl^* mice also showed normal percentages and numbers of thymic compartment subsets compared to littermate controls in 4–6 week old mice (Figure 4D, 4E, Figure S4). We also evaluated T cell development in two-week old mice (since certain phenotypes with ShcA are more evident in younger mice) [40], and observed no obvious differences (Figure S3C). Loss of Shcbp1 did not alter thymocyte survival, as determined by staining for Annexin V and 7AAD in thymocytes freshly isolated from mice, *ex vivo* survival assays, and an *in vivo* dexamethasone injection survival/apoptosis assay (Figure S3E, S3F, S3G). Collectively, these data suggested that, despite a remarkable upregulation of Shcbp1 upon pre-TCR signaling and Shcbp1 correlation with proliferative stages of thymic development, Shcbp1 appears dispensable for T cell development.

One possible reason for the lack of Shcbp1 requirement during thymocyte development is that another protein closely related to Shcbp1 might compensate for its loss. Shcbp1 contains a pectin lyase-like domain (PecLD), which is characterized by a series of parallel β-strands found in enzymes from certain bacterial plant pathogens that digest sugars in the plant wall [4], [41]. Although the role of this domain in mammalian intracellular proteins is unknown, a few other mammalian proteins also contain PecLD sequences including Shcbp1-L (gene name∶*C1ORF14*), Fbox10 (*Fbxo10*), and Fbox11 (*Fbxo11*). When we assessed the expression of genes coding these proteins via RT-PCR, there was no compensatory upregulation in cells lacking Shcbp1 (Figure S2F). Further, the transcript levels of Shcbp1-L and Fbox10 were barely detectable within the thymus. Therefore, other PecLD-containing proteins such as Shcbp1-L, Fbox10, and Fbox11 do not appear to compensate for the loss of Shcbp1.

### Shcbp1 is upregulated by stimulation in peripheral CD4^+^ T cells

**Figure 5:**
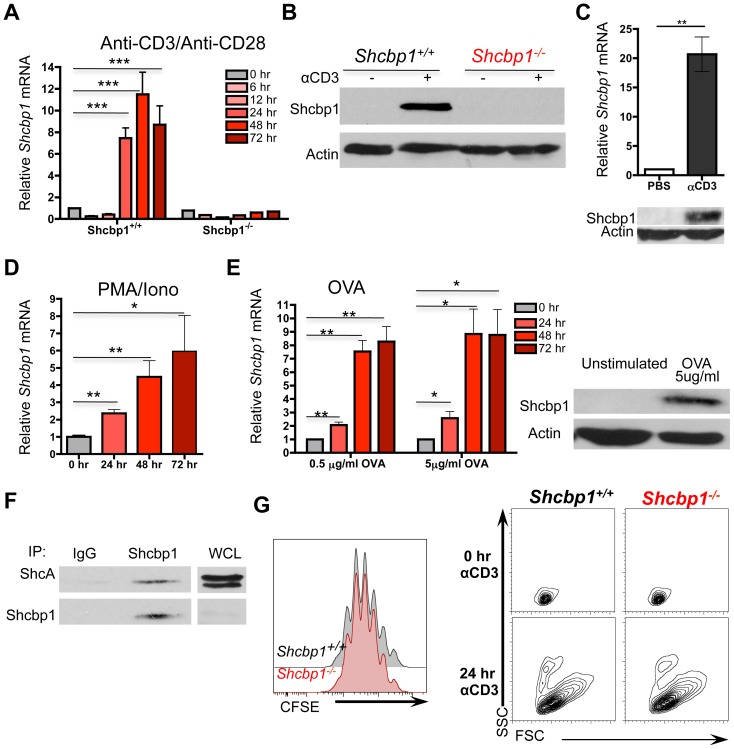
Shcbp1 is upregulated by stimulation in peripheral CD4 T cells. (A) RT-PCR for *Shcbp1* mRNA expression in *ex vivo* anti-CD3/anti-CD28 stimulated CD4^+^ T cells for the indicated times (normalized to *HPRT* and unstimulated CD4^+^ T cells, n = 4 for *Shcbp1^+/+^* and n = 2 for *Shcbp1^−/−^*). (B) Immunoblotting of Shcbp1 from total splenocytes (*Shcbp1^+/+^* or *Shcbp1^−/−^*) stimulated with plate-bound anti-CD3/anti-CD28 for 72 hours (representative of n = 3 experiments). (C) Top, *Shcbp1* mRNA expression in CD4^+^ T cells isolated from wild-type mice injected with either PBS or 100 µg of anit-CD3 for 24 hours (n = 6 mice of each treatment, p = 0.0012). Bottom, representative immunoblot for Shcbp1. (D) *Shcbp1* mRNA in CD4 T cells stimulated with PMA/Ionomycin for the indicated times (normalized to *HPRT* and unstimulated CD4^+^ T cells) (n = 3, * p<0.02). (E) Left, RT-PCR for *Shcbp1* in CD4^+^ T cells isolated from DO11.10 mice stimulated with OVA_(323–339)_ for the indicated times (normalized to *HPRT* and unstimulated CD4^+^ T cells, n = 3 experiments, *p<0.03). Right, representative immunoblot for Shcbp1 (n = 2). (F) Immunoprecipitation of Shcbp1 and immunoblotting of ShcA and Shcbp1 in primary murine splenocytes activated with plate bound anti-CD3/anti-CD28 for 72 hours (representative of n = 2 experiments). (G) Flow cytometry of CD4^+^ T cells isolated from *Shcbp1^+/+^* and *Shcbp1^−/−^* mice, stained with CFSE, and activated with anti-CD3/anti-CD28 for 72 hours (representative of n = 3). Left, representative plot of forward scatter and side-scatter in naïve or stimulated CD4^+^ T cells.

To test whether Shcbp1 is upregulated during activation in peripheral T cells, we performed anti-CD3/anti-CD28 stimulation of CD4^+^ T cells sorted from the lymph nodes. Shcbp1 was upregulated 24 hours after anti-CD3/anti-CD28 stimulation (Figure 5A). We also confirmed Shcbp1 protein upregulation by immunoblotting (Figure 5B). To test whether Shcbp1 was upregulated after T cell activation *in vivo*, we injected anti-CD3 into C57BL/6J mice and found ∼20-fold increase in Shcbp1 transcript and protein levels in CD4^+^ T cells at 24 hours after injection (Figure 5C). We further examined whether Shcbp1 was induced in CD4^+^ T cells activated by stimuli that bypass the TCR and found comparable *Shcbp1* upregulation after PMA and ionomycin stimulation (Figure 5D). To determine whether Shcbp1 is induced by physiological, antigen-specific, TCR stimulation, we used DO11.10 TCR transgenic mice, which express a transgenic TCR that recognizes a peptide containing amino acids 323 through 339 of ovalbumin (OVA_(323–339)_) [42]. Activation of DO11.10 CD4^+^ T cells with two different doses of OVA_(323–339)_ showed that *Shcbp1* was induced by antigen-specific TCR signaling (Figure 5E). Additionally, we confirmed that Shcbp1 binds to ShcA in activated splenocytes (Figure 5F) [1]. Collectively, these results demonstrate that Shcbp1 was induced by antigen-specific, antibody-mediated, and chemical T cell stimulation.

Since Shcbp1 was upregulated after T cell stimulation and T cell development also involves the migration of thymocytes out of the thymus, [43], [44] we next investigated the peripheral T cell compartment. In mice with global or conditional deletion of *Shcbp1*, there were no obvious differences in the fraction or absolute numbers of CD4^+^ and CD8^+^ T cells in the spleen or lymph nodes (Figure 4F, 4G, S5A, S5B). There were also no differences in the expression of the regulatory T cell transcription factor FOXP3 or a panel of markers found on activated or memory cells (Figure S5C, S5D). These data suggested that Shcbp1 was not required for the development or maintenance of the peripheral T cell compartment.

To determine whether Shcbp1 was required for T cell proliferation, we labeled *Shcbp1^+/+^* and *Shcbp1^−/−^* CD4^+^ T cells with the cytosolic dye CFSE and stimulated with anti-CD3/anti-CD28 for various times (24 hours, 48 hours, 72 hours). However, we observed no discernible differences in the proliferation of *Shcbp1^+/+^* and *Shcbp1^−/−^* T cells as monitored by the CFSE dilution (Figure 5G). Further, T cells deficient in *Shcbp1* upregulated markers of T cell activation and became blastic by 24-hours after stimulation (Figure 5G, S5D). These data suggest that Shcbp1 was likely dispensable for *ex vivo* anti-CD3/anti-CD28 mediated T cell proliferation and activation, as measured under these assay conditions.

### Loss of Shcbp1 affects disease severity in CD4^+^ T cell driven autoimmune disease

**Figure 6:**
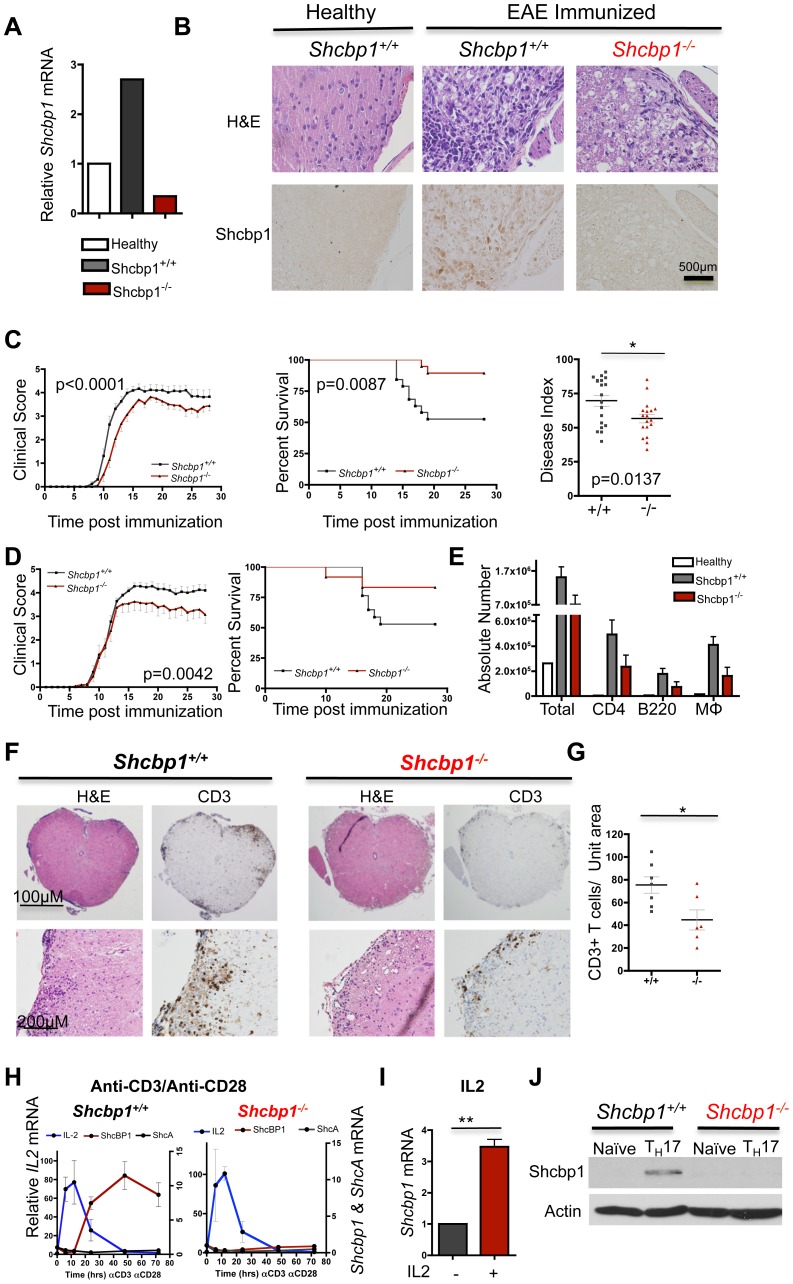
Loss of Shcbp1 affects disease severity in CD4^+^ T cell driven autoimmune disease. (A) *Shcbp1* mRNA expression in mononuclear cells isolated from the brain and spinal cords of healthy controls or from mice immunized with MOG_(35–55)_ on day 28 after disease induction (Normalized to *HPRT* and healthy controls) (n = 3 mice of each condition, pooled). (B) Shcbp1 expression assessed by immunohistochemistry in spinal cords isolated from healthy controls or *Shcbp1^+/+^* and *Shcbp1^−/−^* mice on day 28 after immunization for EAE (n = 3 mice of each condition). (C) Left, mean clinical scores of EAE in male *Shcbp1^+/+^* and *Shcbp1^−/−^* mice. Middle, percent survival of male *Shcbp1^+/+^* and *Shcbp1^−/−^* mice. Right, disease index (area under the curve) plotted for each mouse individually. Compiled data with n = 18–19 mice of *Shcbp1^+/+^* and *Shcbp1^−/−^* genotype, respectively. (D) Left, mean clinical scores of EAE in female *Shcbp1^+/+^* and *Shcbp1^−/−^* mice. Right, percent survival of female *Shcbp1^+/+^* and *Shcbp1^−/−^* mice (n = 17, 12 *Shcbp1^+/+^* and *Shcbp1^−/−^* mice respectively). (E) Left, absolute numbers of total cells, CD4^+^ T cells, B cells, and macrophages isolated from the spinal cords and brain of *Shcbp1^+/+^* and *Shcbp1^−/−^* mice on day 28 after immunization (n = 3 mice of each genotype). (F) Immunohistochemistry for CD3 and H&E staining on sacral spinal cord sections from *Shcbp1^+/+^* and *Shcbp1^−/−^* mice on day 28 after immunization with EAE (representative of n = 7 *Shcbp1^+/+^* and n = 6 *Shcbp1^−/−^* mice, respectively). (G) Quantification of number of CD3^+^ cells from the sacral spinal cord sections (n = 7 and n = 6 for *Shcbp1^+/+^* and *Shcbp1^−/−^* mice, respectively, p = 0.02). (H) Relative expression of *ShcA*, *Shcbp1*, and *IL2* in *Shcbp1^+/+^* T cells (left) or *Shcbp1^−/−^* T cells (right) after activation with anti-CD3/anti-CD28. Normalized to *HPRT* and unstimulated cells (n = 2–3 experiments). (I) Upregulation of *Shcbp1* in CD4^+^ T cells stimulated with IL2 (normalized to *HPRT* and unstimulated cells) (n = 3, p = 0.01). (J) Immunoblotting for Shcbp1 and actin (loading control) in naïve and T_H_17 skewed cells from *Shcbp1^+/+^* and *Shcbp1^−/−^* mice (representative of n = 2 experiments).

Previous research has shown that Shcbp1 protein is upregulated in T cells from *CTLA4*-deficient mice compared to T cells from wild-type mice [1]. *CTLA4*-deficient mice have high lymphoproliferation and lethal autoimmunity [45], [46]. To test whether Shcbp1 might play a role *in vivo* in T cell effector responses in the context of autoimmune disease, we chose to use the CD4 T cell driven experimental autoimmune encephalomyelitis (EAE) model. The EAE model reproduces many of the clinical, pathological, and immunological aspects of the human disease multiple sclerosis (MS), including infiltration of autoreactive T cells into the central nervous system (CNS), causing inflammation and demyelination [47], [48], [49]. Moreover, many genetic loci identified as conferring susceptibility to MS are linked to CD4 effector T cell differentiation and function [50], [51]. We first analyzed the expression of Shcbp1 in the spinal cords of mice immunized with MOG_35–55_ peptide to induce EAE. *Shcbp1* expression was upregulated in mononuclear cells from the spinal cord and brain of animals subjected to EAE (compared to healthy controls) as analyzed via RT-PCR (Figure 6A). Shcbp1 protein expression was also detectable by immunohistochemistry in the spinal cords of EAE mice, especially in the areas of cellular immune infiltration, while it was mostly absent in healthy control animals (Figure 6B).

We next analyzed disease severity in *Shcbp1^−/−^* and control mice after MOG_35–55_ peptide injection to induce disease. Two independent investigators, blinded to the genotype of the mice, monitored clinical scores for a period of 28 days after peptide immunization. *Shcbp1* deficiency consistently resulted in a reduction of disease severity along with improved survival in both male and female mice subjected to EAE (Figure 6C, 6D) (n = 3 independent experiments with a total of n = 18, 19 male and n = 17, 12 female *Shcbp1^+/+^* and *Shcbp1^−/−^* mice, respectively). *Shcbp1* deficient mice exhibited a lower maximum score and reduced overall disease index (area under the curve) (Table 1). On day 28 of the disease, we also examined the composition of the immune infiltrate in the CNS by analyzing the cells isolated from the brain and spinal cord of *Shcbp1^+/+^* and *Shcbp1^−/−^* mice via flow cytometry. *Shcbp1^−/−^* mice displayed a trend towards overall fewer mononuclear cells isolated from the brain and spinal cord compared to *Shcbp1^+/+^* mice (Figure 6E). Although the overall percentage of each cell type was not significantly altered, *Shcbp1^−/−^* mice had fewer CD4^+^ T cells, B cells, and macrophages (Figure 6E). Histological analysis by H&E staining of the spinal cords confirmed that *Shcbp1* deficient mice had fewer loci of immune infiltration (Figure 6F). Importantly, *Shcbp1^−/−^* mice also had significantly fewer CD3^+^ T cells in their spinal cords, as detected by immunohistochemistry (Figure 6F, 6G). Together, these data suggested that Shcbp1 is upregulated in the CNS tissue in the EAE model and that loss of Shcbp1 expression attenuates disease severity.

**Table 1:** Parameters of EAE disease in *Shcbp1^+/+^* and *Shcbp1^−/−^* mice.

Genotype	Incidence	Day of Onset	Mean Max	Disease Index	Mortality
***Shcbp1*** ^+//+^ ** (Males)**	100% (19/19)	10.4±1.2	4.5±0.56	69.6±17.2	47.4% (9/19)
***Shcbp1*** ** (Males)**	100% (18/18)	11.8±1.5**	4.1±0.45*	56.6±13.0*	11.1%** (2/18)
***Shcbp1*** ^+/+^ ** (Females)**	100% 17/17	10.5±1.2	4.5±0.56	70.0±13.9	47% (8/17)
***Shcbp1*** ** (Females)**	100% 12/12	11.5±3.3	3.9±1.0	59.0±21.9	16.6% (2/12)

Data is expressed as mean ± SEM and values shown are compiled results from 3 independent experiments.

*p<0.05,

**p<0.01.

*Rag1^−/−^* mice are resistant to EAE due to the lack of mature T cells, and their engraftment with T cells isolated from the secondary lymphoid organs of wild-type mice has been demonstrated to be sufficient for disease development [52]. To directly test whether T cell specific expression of Shcbp1 contributes to disease severity, we transferred *Shcbp1*-deficient or control T cells into *Rag1^−/−^* mice, and induced disease one week after transfer. The *Rag1^−/−^* deficient mice that received *Shcbp1* deficient CD4^+^ T cells had overall reduced disease severity along with improved survival, compared to *Rag1^−/−^* mice that received wild-type CD4^+^ T cells (Figure S6A, S6B). As an additional control, we immunized mice that received no transfer of CD4^+^ T cells, and confirmed that these mice did not develop disease (Figure S6A, S6B). Furthermore, we immunized *Lck*-Cre^+^/*Shcbp1^fl/fl^* and *Lck*-Cre^+^/*Shcbp1^wt/wt^* mice to induce EAE, and observed reduced disease severity in *Lck*-Cre^+^/*Shcbp1^fl/fl^* mice (Figure S6C). Based on these observations, we conclude that Shcbp1 expression in CD4^+^ T cells contributes to disease severity in the EAE model. However, we cannot exclude the possibility that Shcbp1 expression in cell types other than CD4^+^ T cells may have some contribution to disease severity in the EAE model.

In multiple sclerosis and EAE, there is inappropriate T cell activation as well as abnormal IL-2 and T_H_17 skewing conditions within the cerebrospinal fluid (CSF) and serum [53], [54], [55]. Therefore, we next determined whether the conditions likely present in the CSF in MS and EAE were capable of upregulating Shcbp1 expression in CD4^+^ T cells. We evaluated how the kinetics of Shcbp1 upregulation (Figure 5A) correlated with upregulation of IL-2 after anti-CD3/anti-CD28 stimulation. *IL-2* was upregulated rapidly after stimulation, with maximal induction around 12 hours post-stimulation, while *Shcbp1* upregulation occurred with much delayed kinetics (Figure 6H). Since *IL-2* was upregulated prior to *Shcbp1* induction, we also tested whether IL-2 could induce Shcbp1. IL-2 stimulation induced *Shcbp1* expression, suggesting that Shcbp1 is a novel IL-2 responsive gene (Figure 6I). We also found that Shcbp1 was upregulated after culturing CD4^+^ T cells in T_H_1 or T_H_17 skewing conditions (Figure 6J, S6 D-F). Therefore, conditions that are present and therapeutically relevant in multiple sclerosis and EAE were capable of inducing *Shcbp1* expression in CD4^+^ T cells *ex vivo*.

## Discussion

Recently, several laboratories have identified Shcbp1 through unbiased screening techniques and have linked Shcbp1 to diverse biological functions including embryonic development, cytokinesis, spermatogenesis, growth factor signaling, neuronal development, viral responses, and tumorigenesis [1], [4], [5], [6], [7], [8], [9]. Remarkably, many of these processes have links to proliferation. While these data were suggestive, none of the previous studies evaluated the *in vivo* requirement for Shcbp1. In this report, by generating mouse strains with conditional and global deletion of *Shcbp1*, we have carefully evaluated the *in vivo* requirement for Shcbp1 in T cell development as well as in an autoimmune disease model.

Although the primary objective of our work was to determine the function of Shcbp1 in the T lymphocyte lineage, a significant unexpected finding was that Shcbp1 is dispensable for embryonic development in mice. Since previous studies have shown that deletion of the Drosophila homolog Nessun Dorma resulted in partial lethality and Shcbp1 is highly expressed in the embryo [1], [4], it was possible that Shcbp1 may also be required for embryonic development in the mammalian system [6]. Additionally, Shcbp1 is highly expressed in the testis and ovaries, and male Drosophila lacking Nessun Dorma are not fertile [1], [4]. However, our findings did not support a role for Shcbp1 in mouse spermatogenesis or oogenesis *in vivo*, as both male and female *Shcbp1^−/−^* mice are fertile. Thus, surprisingly, *Shcbp1* is not an essential gene in the mouse, despite significant similarity between the mouse and fly proteins, and these mice do not show compensatory upregulation of proteins with known similarity.

Given the high expression of Shcbp1 in the thymus and activated T cells, we also determined the function of Shcbp1 in the T lymphocyte lineage. Proliferation during T cell development is tightly regulated and thymocytes undergo stages of active proliferation followed by temporary withdrawal from the cell cycle [16]. Shcbp1 expression tightly correlated with actively proliferating thymic subsets and was upregulated via optimal ShcA-mediated preTCR signaling. However, despite the striking correlation of Shcbp1 and proliferative stages of thymocyte development, there was no apparent defect in thymocyte development *in vivo* in the absence of Shcbp1.

Interestingly, we found that Shcbp1 plays a role in CD4^+^ T cells in the context of the autoimmune EAE model of multiple sclerosis. Shcbp1 expression was induced in the brain and spinal cords of mice immunized for EAE and Shcbp1 expression was co-localized with immune infiltrates of the spinal cord. Previous studies have shown that cytokines, including IL2 and the T_H_17 skewing cytokines (IL23, IL6, TGFβ), are abnormally present in the CSF of some patients with multiple sclerosis as well as in mice immunized for EAE [53], [54], [55], [56]. Further, mutations within the IL2 promoter and IL2-receptor confer susceptibility to MS [50], [57], [58], and targeting the IL2 signaling pathway via IL2-receptor blocking antibodies is therapeutically beneficial in humans [59]. Similarly, *IL2*-deficient mice are resistant to EAE induction [60]. Additionally, multiple components of the T_H_17 cell differentiation pathway (IL6, STAT3, IL21) are located near loci that have been identified to confer susceptibility to MS [50] and targeting components of T_H_17 cell differentiation and function has also been shown to confer resistance or lead to attenuated disease in EAE [61], [62]. Therefore, Shcbp1 is induced in T cells under conditions that are likely present and functionally relevant in the inflammatory environment of the CNS during MS/EAE. Furthermore, we found that mice deficient in Shcbp1 had overall reduced EAE disease severity as well as markedly improved survival compared to wild-type control mice, likely due to impairment in the *in vivo* effector function of the CD4^+^ T cells.

The current treatment approaches for multiple sclerosis and many other autoimmune diseases involve the use of immunosuppressant drugs that broadly suppress the immune system with many adverse side-effects including susceptibility to infections and cancers [47], [48]. Recent reports have highlighted the potential benefits of new therapies that specifically target the encephalitogenic T cells during EAE and, by extension, multiple sclerosis [63]. Given the expression of Shcbp1 in inflammatory lesions within the spinal cord as well as reduced disease severity in Shcbp1 deficient mice, Shcbp1 may represent a therapeutic target for autoimmune disease such as multiple sclerosis. Additionally, our findings suggest that targeting Shcbp1 may have an additional benefit of minimal side effects, since loss of Shcbp1 does not appear to directly affect development or proliferation of CD4^+^ T cells.

## Materials and Methods

### Ethics statement

All animal experiments conducted in this study were carried out in strict accordance with protocols approved by the University of Virginia Animal Care and Use Committee (Protocol number: 2992). All experiments followed the recommendations in the Guide for the Care and Use of Laboratory Animals of the National Institutes of Health (OLAW/NIH, 2002) and followed the requirements of the Animal Welfare Act (Public Law 91-579). All efforts were made to minimize animal suffering including the use of anesthesia (isoflurane delivered at 5% for induction and 3% for maintenance in oxygen in a precision vaporizer) for the immunizations and use of humane endpoints for the EAE experiments (as detailed below in EAE section). Mice were monitored 2-times daily and were euthanized, as necessary, via a carbon dioxide chamber followed by confirmation via cervical dislocation. These methods are consistent with the recommendations of the Panel on Euthanasia and approved by the UVA Animal Care and Use Committee.

### Mice

JM8.A2 embryonic stem cells (C57BL/6N origin) carrying *loxP* sites flanking exons 4–6 of the *Shcbp1* locus were obtained from EUCOMM and injected into C57BL/6J blastocysts by the University of Virginia Gene Targeting and Transgenic Core Facility [34], [64]. The resulting chimeric founder mice were mated to C57BL/6J for germline transmission and the resulting progeny were screened for *Shcbp1* targeting by genotyping for the neomycin targeting cassette. Crossing these to a flippase transgenic mouse to remove the neomycin cassette resulted in mice with floxed *Shcbp1* allele [65]. The *Shcbp1* ^fl/fl^ mouse was subsequently crossed to the *Lck*-Cre transgenic mouse, *Rag*-Cre transgenic mouse, and *EIIA*-Cre transgenic mouse to generate mice with *Shcbp1* conditionally deleted and *Shcbp1* null mice (*Shcbp1^−/−^* mice), respectively [35], [38], [39]. Genotyping for *Shcbp1* wild-type, floxed, and deleted allele was performed on tail DNA in the same reaction using the following primers:

(3′CCACTTGCCCAGGTCAAACTGTAAAA 5′), (3′ATCCTCTTGGAGATCAATCAAATGTTTGTG 5′), and (3′GAGAGGAGATGTATGTATTTGTTGAACTGATGG 5′).

All mice used were on the C57BL/6J background unless otherwise noted. Wild type C57BL/6J mice, *Rag1* deficient mice, *Lck*-Cre, *EIIA*-Cre and flippase transgenic mice were purchased from the Jackson Laboratories or Taconic [35], [38], [66]. The *Lck*-Cre/ShcFFF and *Lck*-Cre/ShcFF mouse lines have been previously described [30]. The *Rag*-Cre (mixed background) transgenic mouse line and the DO11.10 TCR transgenic (BALB/c) were kindly provided by the Bender and Lorenz laboratories, respectively, at the University of Virginia [39], [42]. Mice were bred and housed under specific pathogen-free environment in a 12 hour light-dark cycle with ad libitum access to food and water and all efforts were made to minimize animal suffering. The ‘n’ was determined using power calculations (G*Power 3) that account for the statistical analysis chosen (ANOVA test or t-test) and anticipated variability, which is estimated based on similar experiments performed previously. Shcbp1^fl/fl^ mice are available through The Jackson Laboratory as Stock No. 025770.

### Flow Cytometry

Thymocytes, splenocytes, and lymphocytes were isolated from 4- to 6-week old mice (littermates) for analysis of T cell development and activation. DP and DN compartments were analyzed by staining thymocytes with antibodies specific for mouse CD4, CD8, CD3, Thy1.2, CD25, CD44, CD28, TCRβ, and TCRγδ as well as lineage markers (CD11b, CD11c, B220, GR1, Ly6G, and Ter119) at 1∶100 dilution as described previously [22]. Absolute numbers were determined via enumeration with the hemocytometer followed by flow cytometry analysis. Viability and apoptosis were evaluated by staining with Annexin V and 7AAD (Invitrogen), according to manufacturer's instructions. Splenocytes and lymphocytes were stained with antibodies specific for CD3, CD4, CD8, FoxP3, as well as CD62L, CD44, CD25, and CD69. All antibodies used were obtained from eBioscience unless otherwise noted. FACSCanto (BD Biosciences) was used for flow cytometry and results were analyzed by FlowJo software (TreeStar Inc.).

### T cell stimulation, proliferation, and skewing

For anti-CD3/anti-CD28 stimulation, 80,000 CD4^+^ MACS selected T cells (Miltenyi Biotec) were stimulated with anti-CD3/anti-CD28 beads (Dynabeads, Life Technologies) according to the manufacturer's protocol. To assess proliferation via CFSE dilution, T cells were stained with 5 µM CFSE (Molecular Probes) prior to stimulation [67]. OVA peptide stimulation was performed by plating 40,000 DO11.10 TCR transgenic CD4^+^ T cells along with 80,000 mitomycin (Sigma)-treated APCs and the indicated concentration of OVA_(323–339)_ peptide (AnaSpec). Stimulations were also performed by culturing cells with 50 ng/ml PMA (phorbol 12-myristate 13-acetate; Calbiochem) along with 500 ng/ml ionomycin (Calbiochem). All stimulations were performed in 200 µl RPMI 1640 medium (supplemented with 10% FBS, 50 µM β-Mercaptoethanol, 2-mM L-glutamine, and 1% pennicillin/streptomycin) in round bottom 96-well plates and cultured at 5% CO_2_ at 37°C. Alternatively, T cell stimulations were performed in 24-well plates (Costar) using 2 µg/ml plate-bound anti-CD3 (BD Pharmingen) and either 1 µg/ml soluble anti-CD28 (BD Pharmingen) or 100 U/ml IL-2 (Peprotech).

*In vivo* T cell stimulation was performed by intraperitoneal injection of 100 µg of anti-CD3. CD4^+^ T cells were harvested from the spleen and lymph nodes 24 hours after injection. For the *in vivo* model of preTCR signaling, *Rag1*-deficient mice were injected with 100 µg of anti-CD3 intraperitoneally and the thymus was collected 24 hours after injection [33].

T_H_17 skewing was performed by selecting naïve CD4^+^ CD62L^+^ T cells from spleens and lymph nodes of 4-week old mice (Miltenyi Biotec). Naïve T cells were skewed for 4 days on 1 µg/ml anti-CD3 and 2 µg/ml anti-CD28 coated plates along with 0.3 ng/ml TGF-β1 (R&D Systems), 20 ng/ml IL-6 (R&D Systems), 10 ng/ml IL-23 (eBioscience), 10 µg/ml anti-IL4 (eBioscience), and 10 µg/ml anti-IFNγ (eBioscience) in IMDM supplemented with 10% FBS, 50 µM β-Mercaptoethanol, 2-mM L-glutamine, non-essential amino acids, 1 mM sodium pyruvate, and 10 mM Hepes. After 4 days, cells were collected for analysis. Cells analyzed by intracellular cytokine staining were stimulated with 50 ng/ml PMA and 1 µM Ionomycin along with GolgiStop (BD Pharmingen) for 6 hours prior to staining. Intracellular staining for IL-17A (BD) and IFNγ was performed by fixing the cells in 4% paraformaldehyde followed by permeabilization with 0.1% Saponin. T_H_1 skewing was performed by skewing naïve CD4^+^ CD62L^+^ T cells on 1 µg/ml anti-CD3 and 2 µg/ml anti-CD28 coated plates along with 100 U/ml IL-2 (Peprotech), 10 ng/ml IL-12 (Ebiosciences), and 10 µg/ml anti-IL4 (eBioscience) and analysis was performed on day 7 as described above.

### Experimental Autoimmune Encephalomyelitis

EAE immunization was performed as previously described [68]. In brief, 10 week-old female or male mice were anesthetized with isoflurane and immunized subcutaneously into the upper and lower back with 200 µg MOG_35–55_ peptide (CS Bio Co), emulsified in equal volume of complete Freund's adjuvant (Sigma) supplemented with heat killed M. tuberculosis (clone H37RA) (Difco) for a total of 400 µg H37RA per mouse. Mice received 200 ng of pertussis toxin (List Biologicals) intraperitoneally on day 0 and 1 after immunization. Two investigators, blinded to the genotype of mice, independently analyzed the mice daily on a 5-point scale: 0-no clinical signs; 1-paralyzed tail; 2-mild hindlimb paresis; 3-severe hindlimb paresis; 4-hindlimb paralysis; 5 quadriplegia/moribund. Mice were weighed and monitored two-times daily by the investigators and were also monitored once-daily by the animal care technicians and, as necessary, the veterinarians. Mice were euthanized at humane endpoints; mice that had a loss of 20% bodyweight or exhibited immobility/quadriplegia were euthanized via a carbon dioxide chamber followed by cervical dislocation to minimize suffering. To minimize distress, mice with hindlimb dysfunction/paralysis received special bedding, soft food, and were separated from any healthy mice.

Brain and spinal cord leukocytes were isolated on day 28 post-injection using Percoll (GE Healthcare) gradient centrifugation, according to published protocols. Isolated cells were identified by staining with antibodies specific for CD4, CD45, CD11b, and B220 followed by flow cytometry. For histological analysis, mice were perfused with 4% paraformaldehyde and the sacral, lumbar, thoracic, and cervical parts of the spinal cord were fixed in 4% paraformaldehyde and embedded in paraffin.

For CD4^+^ T cell transfer EAE experiments, while under isoflurane anesthesia *Rag1^−/−^* mice were given via retro-orbital injection, 2×10^6^ CD4^+^ selected T cells from either *Shcbp1^+/+^* or *Shcbp1^−/−^* mice. One week after CD4^+^ T cell transfer, mice were immunized for EAE and scored as described above.

### Quantitative PCR

Total RNA was extracted from thymocytes and selected CD4^+^ T cells using a QIAshredder and RNeasy kit (Qiagen) followed by reverse transcription using the SuperScript III (Invitrogen) kit. Quantitative PCR was performed using the TaqMan Gene Expression assays (Applied Biosystems) on a StepOnePlus system (Applied Biosystems). TaqMan gene expression probes were used for gene analysis of mouse *Shcbp1*, *ShcA*, *HPRT*, *Shcbp1-L*, *Fbox10*, *Fbox11* and *IL-2*. Each sample was performed in duplicate, target transcripts were normalized to *HPRT* mRNA as an internal control gene, and the relative expression of each target gene was calculated using the comparative cycling method with StepOne v2.1 software (Applied Biosystems).

### Immunohistochemistry and Immunofluorescence

For detection of Shcbp1 *in vivo*, thymi were fixed by immersion in 10% neutral buffered formalin (Fisher) and embedded in paraffin blocks. Sections were processed for immunohistochemistry using standard techniques. Briefly, Shcbp1 staining was performed using a purified rabbit Shcbp1 polyclonal antibody at 1∶500 (Schmandt *et al*) followed by amplification using the Vectastain ABC kit (Vector laboratories); the peroxidase detection was performed using the DAB peroxidase substrate kit (Vector Laboratories). Images were acquired on an Olympus SZX12 low magnification microscope equipped with an Olympus DP70 digital camera.

For detection of Shcbp1 via immunofluorescence, thymi were embedded in OCT (Torrance) and frozen at −80°C. Frozen sections were cut to 4 µm in thickness and fixed in 4% paraformadehyde followed by permeabilization in 0.1% Triton. Frozen sections were rehydrated with PBS, blocked for 60 minutes with 5% (vol/vol) goat serum, and stained overnight at 4°C with the Shcbp1 specific antibody (Schmandt *et al*) at 1∶100. Slides were washed with PBS and incubated with appropriate secondary antibody at 1∶300. Slides were further stained with fluorophore-conjugated antibodies specific for CD25, CD4 or CD8 as well as Hoechst (Molecular Probes), and mounted with Prolong Gold antifade reagent (Molecular Probes). Slides were viewed using the Axio Imager 2 with Apotome (Zeiss) and AxioVision software was used for analysis.

### Immunoblotting and Immunoprecipitation

Immunoblotting of primary murine tissues was performed by lysing cells in RIPA buffer containing protease inhibitors (Calbiochem), followed by analysis via SDS-PAGE and immunoblotting. To detect Shcbp1, the following antibodies were used: Shcbp1 rabbit polyclonal (Schmandt *et al*), polyclonal goat PAL N17 (Santa Cruz), polyclonal goat PAL K20 (Santa Cruz), and polyclonal rabbit Shcbp1 C-terminal (Abgent). Immunoblotting for actin (Sigma) was performed on the same blot as a loading control. Quantification was performed using NIH Image-J software.

Immunoprecipitation was performed by lysing thymocytes or the SCB29 cell line [69]. Lysates were incubated with 4 µg anti-Shcbp1 (PAL N17, Santa Cruz) or 4 µg normal goat IgG (Santa Cruz) overnight, followed by an additional 2 hours incubation with protein A/G beads (Santa Cruz). Beads were washed and eluted by boiling in SDS sample buffer containing β-ME and analyzed via SDS-PAGE and immunoblotting for Shcbp1 (Santa-Cruz) and ShcA (BD).

### Cell Culture

Primary murine T cells were cultured as described previously [22]. The SCID murine thymocyte-derived cell line SCB29 has been described previously and was cultured in IMDM supplemented with 10% FBS, 2-mM L-glutamine, and antibiotics at 5% CO_2_ and 37°C [69].

### *In vivo* and *Ex vivo* survival assays

For the *in vivo* model of survival and apoptosis, 4 to 6-week old mice were injected intraperitoneally with 250 µg dexamethasone (Calbiochem). Thymocytes were collected 5 hours post dexamethasone injection and stained with Annexin-V, 7AAD (Invitrogen), and antibodies specific for CD4, CD8, and CD3 and analyzed by flow cytometry. *Ex vivo* survival assays were performed by incubating thymocytes in complete RPMI at 37°C for the indicated times.

### Statistical analysis

Statistical comparisons were performed using student's two-tailed *t*-test or a two-way ANOVA (clinical scores for EAE) using GraphPad Prism version 4.0. Results with a *p*-value lower than 0.05 were considered significant.

## Supporting Information

Figure S1 Viability, gross development, and fertility are normal in *Shcbp1^−/−^* deficient mice. (A) Chart of mice born with the indicated genotypes from a cross of *Shcbp1^+/−^* to *Shcbp1^+/−^*. (B) Weight of 2-week old pups and 10-week old female mice of *Shcbp1^+/+^* and *Shcbp1^−/−^* mice. (C) Chart of the percentage fertile male and female *Shcbp1^+/+^* and *Shcbp1^−/−^* mice.
(TIF)

Figure S2 Generation of *Lck*-Cre^+^/*Shcbp1^fl/fl^* mice and verification of deletion of Shcbp1 in the thymus. (A) PCR for *Shcbp1* WT, *Shcbp1* floxed, and *Shcbp1* deleted loci in thymic DNA, or (B) DNA from sorted T cells and non-T cell splenocytes. (C) *Shcbp1* mRNA in mice with indicated genotypes normalized to *HPRT* and to control mice (n>3 mice per genotype). (D-E) Immunoblots of Shcbp1 from mice with the indicated genotypes. (F) mRNA levels of indicated genes in WT and thymocytes lacking Shcbp1 (n = 5, 6 mice).
(TIF)

Figure S3 Normal development and survival of *Shcbp1* deficient thymocytes. (A) Staining for TCRβ on different thymic subsets from *Shcbp1* WT and deficient thymocytes (n = 2 mice per genotype). (B) Staining for TCRβ and TCRγδ on thymocyte isolated from WT and *Shcbp1*-deficient mice (n = 2 mice per genotype). (C) Flow cytometry for CD4 and CD8 of thymi (top) and spleen (bottom) isolated from 2 week old *Lck*-Cre^+^/*Shcbp1^wt/wt^* and *Lck*-Cre^+^/*Shcbp1^fl/fl^* mice (n = 3–5 mice per genotype). (D) H&E staining of paraffin imbedded thymic sections from *Lck*-Cre^+^/*Shcbp1^wt/wt^* and *Lck*-Cre^+^/*Shcbp1^fl/fl^* mice (representative of n = 2 mice per genotype). (E) Annexin V and 7AAD staining of freshly isolated thymocytes (n = 2 mice per genotype). (F) Quantification of flow cytometric analysis of Annexin V and 7AAD in thymocytes freshly isolated or incubated at 37^o^ for the indicated time (n>3 mice per genotype). (G) Left, schematic of the *in vivo* model of thymic survival and apoptosis. Right, percentage of Annexin V^+^ 7AAD^−^ thymocytes after injection with either PBS or 250 µg dexamethasone (n = 4 mice of each genotype).
(TIF)

Figure S4 Generation and analysis of T cell development in the *Rag*-Cre^+^/*Shcbp1^fl/fl^* mice. (A) Strategy detailing the generation of mice with *Shcbp1* conditionally deleted using the *Rag*-Cre. (B) *Shcbp1* mRNA levels in thymocytes from *Rag*-Cre^+^/*Shcbp1^wt/w^* ^t^ and *Rag*-Cre^+^/*Shcbp1^fl/fl^* mice normalized to *HPRT* and to control (n>3 mice per genotype) (C) Immunoblotting of Shcbp1 in total thymocytes (n = 2 experiments). (D) Flow cytometric analysis of thymi isolated from 4-to-6 week old *Rag*-Cre^+^/*Shcbp1^wt/wt^* and *Rag*-Cre^+^/*Shcbp1^fl/fl^* mice. Top panel shows surface marker expression of CD4 and CD8. Bottom panel depicts surface marker expression of CD44 and CD25 gated on DN thymocytes (CD4^−^ CD8^−^ B220^−^ Gr1^−^ Ter119^−^ CD11b^−^ CD11c^−^) (n = 3–6 mice per genotype, age-matched littermate controls). (E) Total cellularity and absolute number of thymic subsets in 4-to 6-week-old *Rag*-Cre^+^/*Shcbp1^wt/wt^* and *Rag*-Cre^+^/*Shcbp1^fl/fl^* mice (n = 4–6 mice of each genotype with age-matched littermate controls). (F) Flow cytometric analysis for cell surface markers CD4 and CD8 in spleens isolated from 4–6 week old *Rag*-Cre^+^/*Shcbp1^wt/wt^* and *Rag*-Cre^+^/*Shcbp1^fl/fl^* mice (representative of n = 3–6 mice of each genotype, littermate controls).
(TIF)

Figure S5 Peripheral compartment and activation of *Shcbp1^+/+^* and *Shcbp1^−/−^* CD4^+^ T cells. (A) Surface staining, and (B) absolute numbers of CD4^+^ and CD8^+^ cells in spleen and lymph nodes of wild-type and *Shcpb1* deficient mice (n>3 mice per genotype). (C) Intracellular staining for *Foxp3* in CD4^+^ T cells from *Shcbp1* WT and deficient mice (n = 2 mice per genotype). (D) Flow cytometry for cell surface markers (CD44, CD62L, CD25, and CD69, CD4) of CD4^+^ T cells isolated from *Shcbp1^+/+^* and *Shcbp1^−/−^* mice after 24 hour stimulation with anti-CD3/anti-CD28 (n = 3 mice of each genotype).
(TIF)

Figure S6 Shcbp1 expression specifically in T cells contributes to EAE disease severity. (A-B) Survival curves and clinical scores of *Rag1* ^−/−^ mice after EAE induction one-week post transfer with CD4^+^ T cells isolated from either *Shcbp1^−/−^* or *Shcbp1^+/+^* mice (n = 7, 8). (C) Clinical scores of *Lck*-Cre^+^/*Shcbp1^wt/wt^* and *Lck*-Cre^+^/*Shcbp1^fl/fl^* mice after EAE induction (n = 4,8). (D) RT-PCR for *Shcbp1* in naïve or *ex vivo* T_H_17 or T_H_1 skewed CD4^+^ T cells (normalized to *HPRT* and unstimulated CD4^+^ T cells) (n = 2 mice of each genotype) (E-F) Intracellular staining for IL17-A or IFNγ in CD4^+^ T cells from *Shcbp1^+/+^ and Shcbp1^−/−^* mice after skewing (representative of n = 4 experiments with n = 4 mice of each genotype). (G) Cell surface staining for CD11b, CD45, and CD4 in mononuclear cells isolated from healthy controls or *Shcbp1^+/+^* or *Shcbp1^−/−^* mice 28 days after EAE induction.
(TIF)

Figure S7 Original images and gels from all figures and supporting files. This supporting figure includes the original images and gels from all figures and supporting files. The images are not altered in any way and are unmodified and not cropped.
(TIF)

Checklist S1 ARRIVE Guidelines Checklist. Attached is the ARRIVE Guideline checklist for reporting *in vivo* experiments.
(PDF)
