# Supplementary figures and images for: Unexpected Phenotype of Mice Lacking Shcbp1, a Protein Induced during T Cell Proliferation
Source: PLoS One. 2014 Aug 25;9(8):e105576. doi: 10.1371/journal.pone.0105576 (PMC4143286; doi:10.1371/journal.pone.0105576)

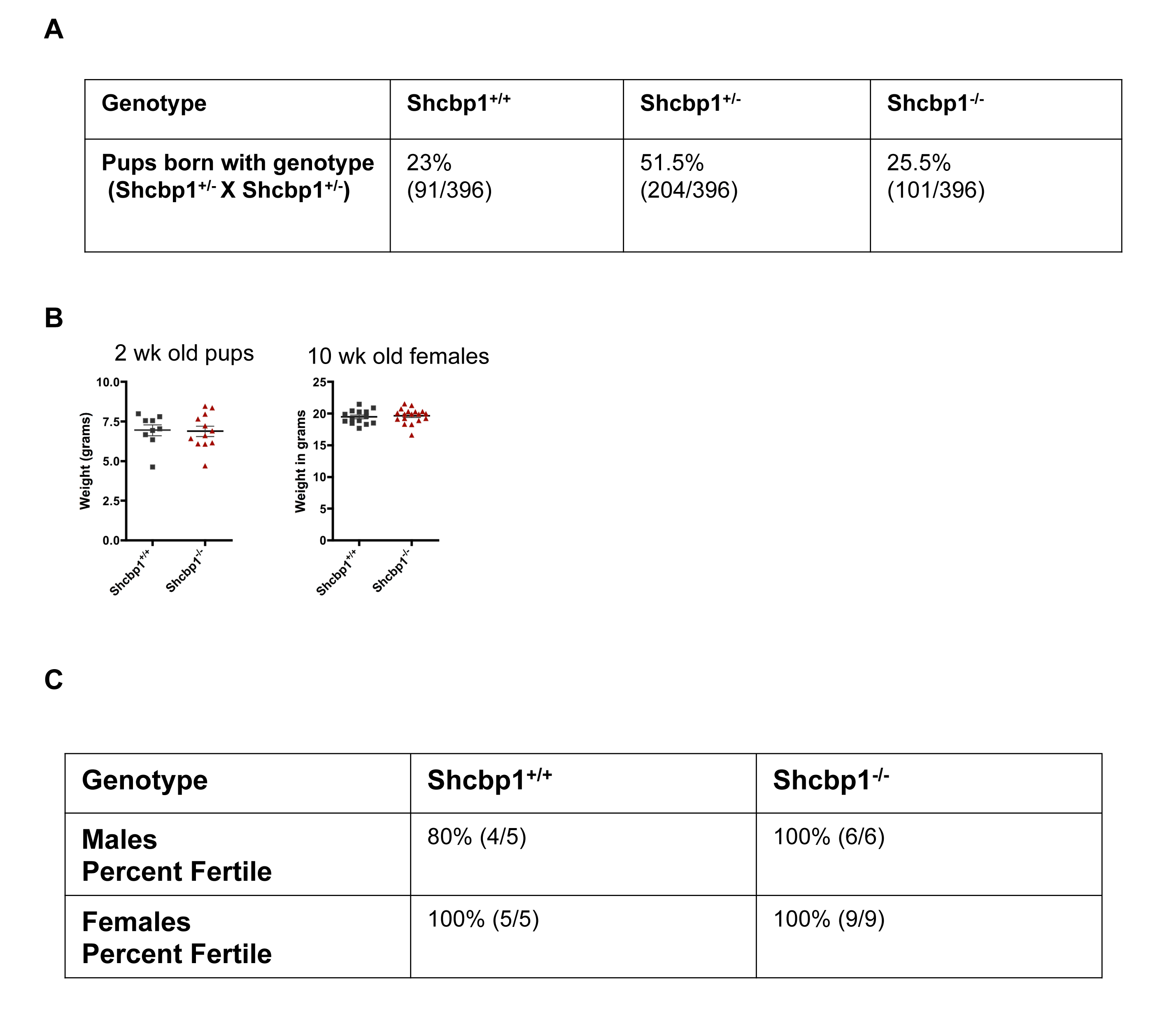

Supplement: Figure S1 — Viability, gross development, and fertility are normal in Shcbp1−/− deficient mice. (A) Chart of mice born with the indicated genotypes from a cross of Shcbp1+/− to Shcbp1+/−. (B) Weight of 2-week old pups and 10-week old female mice of Shcbp1+/+ and Shcbp1−/− mice. (C) Chart of the percentage fertile male and female Shcbp1+/+ and Shcbp1−/− mice. (TIF) [file pone.0105576.s001.tif]

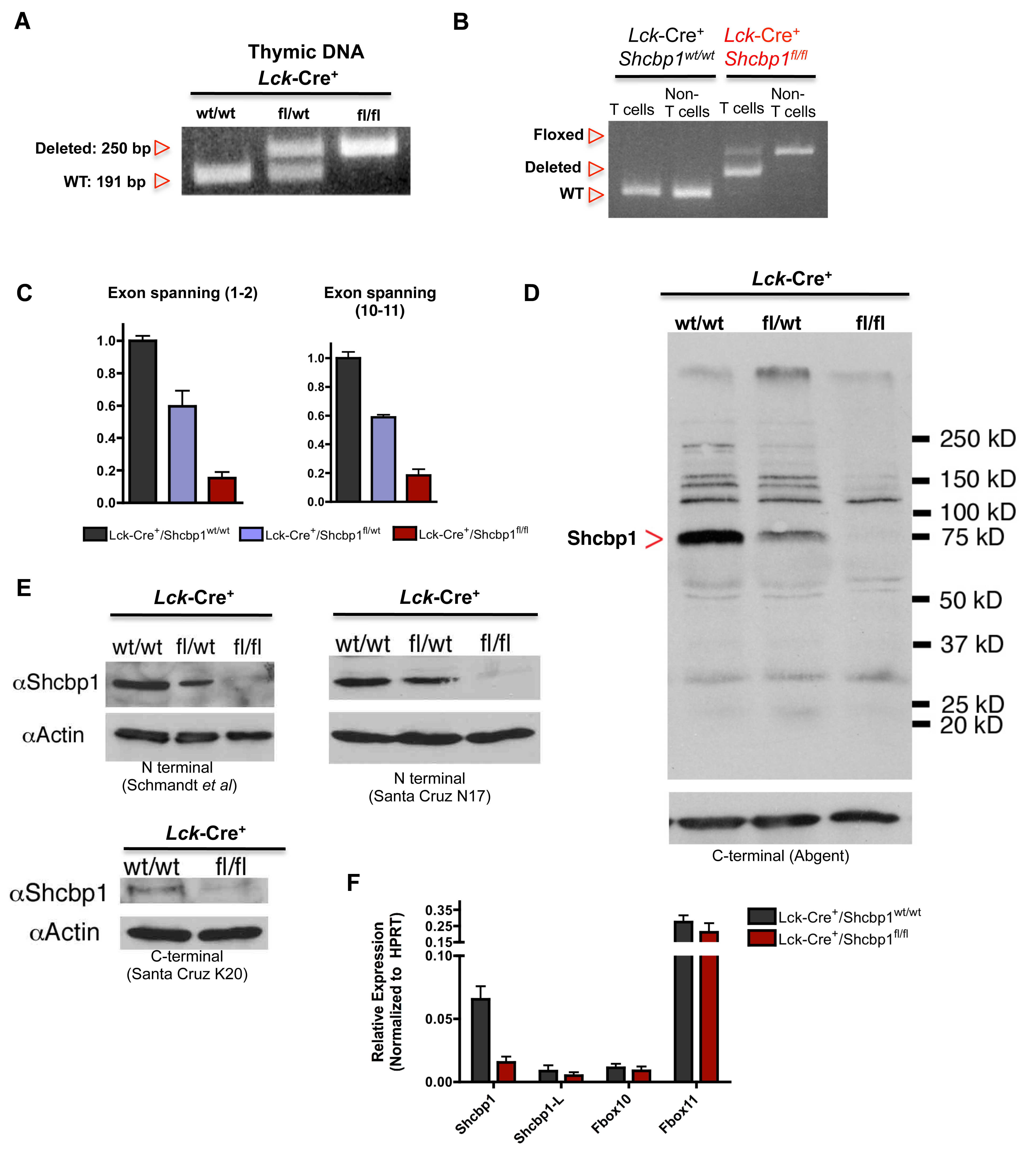

Supplement: Figure S2 — Generation of Lck-Cre+/Shcbp1fl/fl mice and verification of deletion of Shcbp1 in the thymus. (A) PCR for Shcbp1 WT, Shcbp1 floxed, and Shcbp1 deleted loci in thymic DNA, or (B) DNA from sorted T cells and non-T cell splenocytes. (C) Shcbp1 mRNA in mice with indicated genotypes normalized to HPRT and to control mice (n>3 mice per genotype). (D-E) Immunoblots of Shcbp1 from mice with the indicated genotypes. (F) mRNA levels of indicated genes in WT and thymocytes lacking Shcbp1 (n = 5, 6 mice). (TIF) [file pone.0105576.s002.tif]

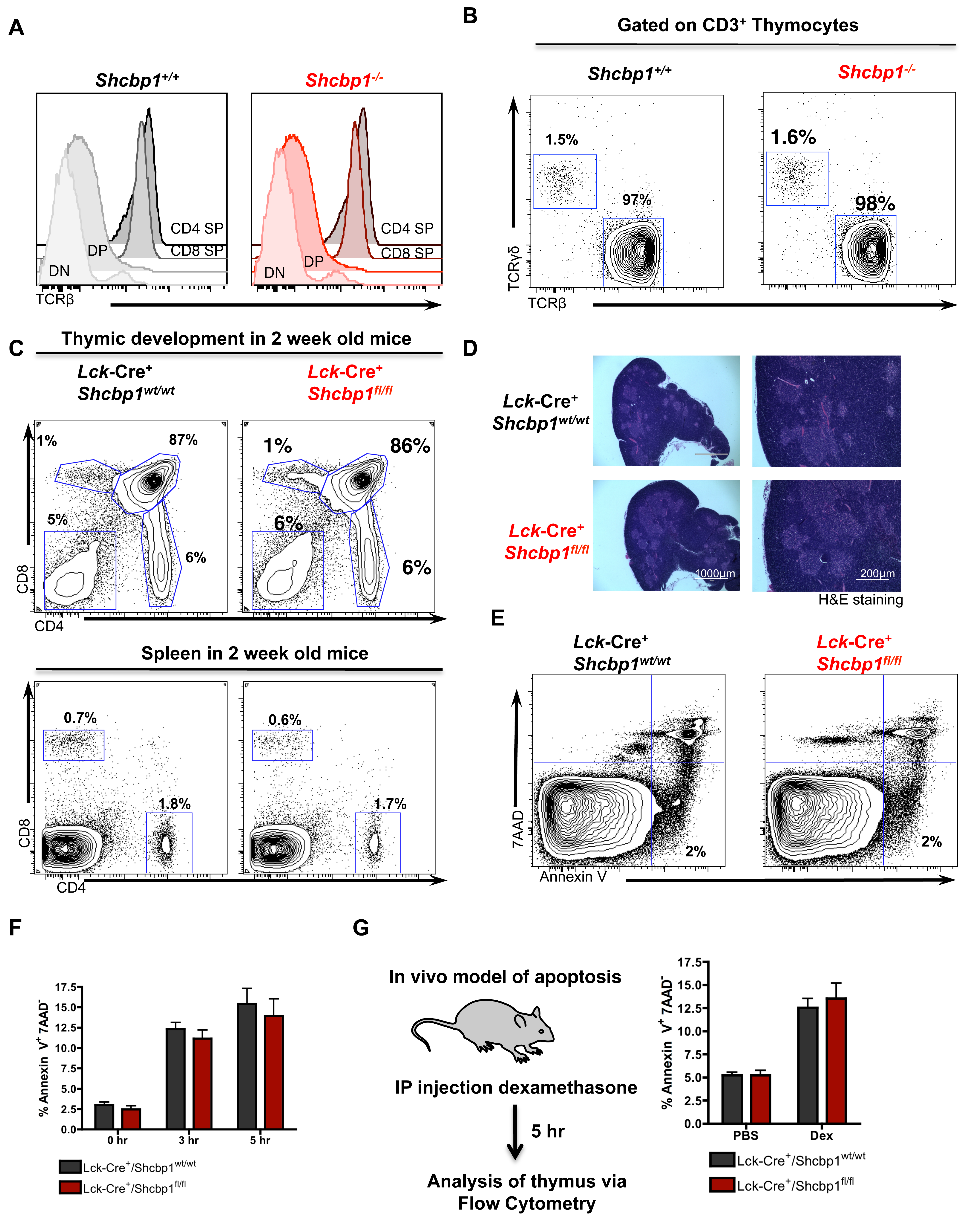

Supplement: Figure S3 — Normal development and survival of Shcbp1 deficient thymocytes. (A) Staining for TCRβ on different thymic subsets from Shcbp1 WT and deficient thymocytes (n = 2 mice per genotype). (B) Staining for TCRβ and TCRγδ on thymocyte isolated from WT and Shcbp1-deficient mice (n = 2 mice per genotype). (C) Flow cytometry for CD4 and CD8 of thymi (top) and spleen (bottom) isolated from 2 week old Lck-Cre+/Shcbp1wt/wt and Lck-Cre+/Shcbp1fl/fl mice (n = 3–5 mice per genotype). (D) H&E staining of paraffin imbedded thymic sections from Lck-Cre+/Shcbp1wt/wt and Lck-Cre+/Shcbp1fl/fl mice (representative of n = 2 mice per genotype). (E) Annexin V and 7AAD staining of freshly isolated thymocytes (n = 2 mice per genotype). (F) Quantification of flow cytometric analysis of Annexin V and 7AAD in thymocytes freshly isolated or incubated at 37o for the indicated time (n>3 mice per genotype). (G) Left, schematic of the in vivo model of thymic survival and apoptosis. Right, percentage of Annexin V+ 7AAD− thymocytes after injection with either PBS or 250 µg dexamethasone (n = 4 mice of each genotype). (TIF) [file pone.0105576.s003.tif]

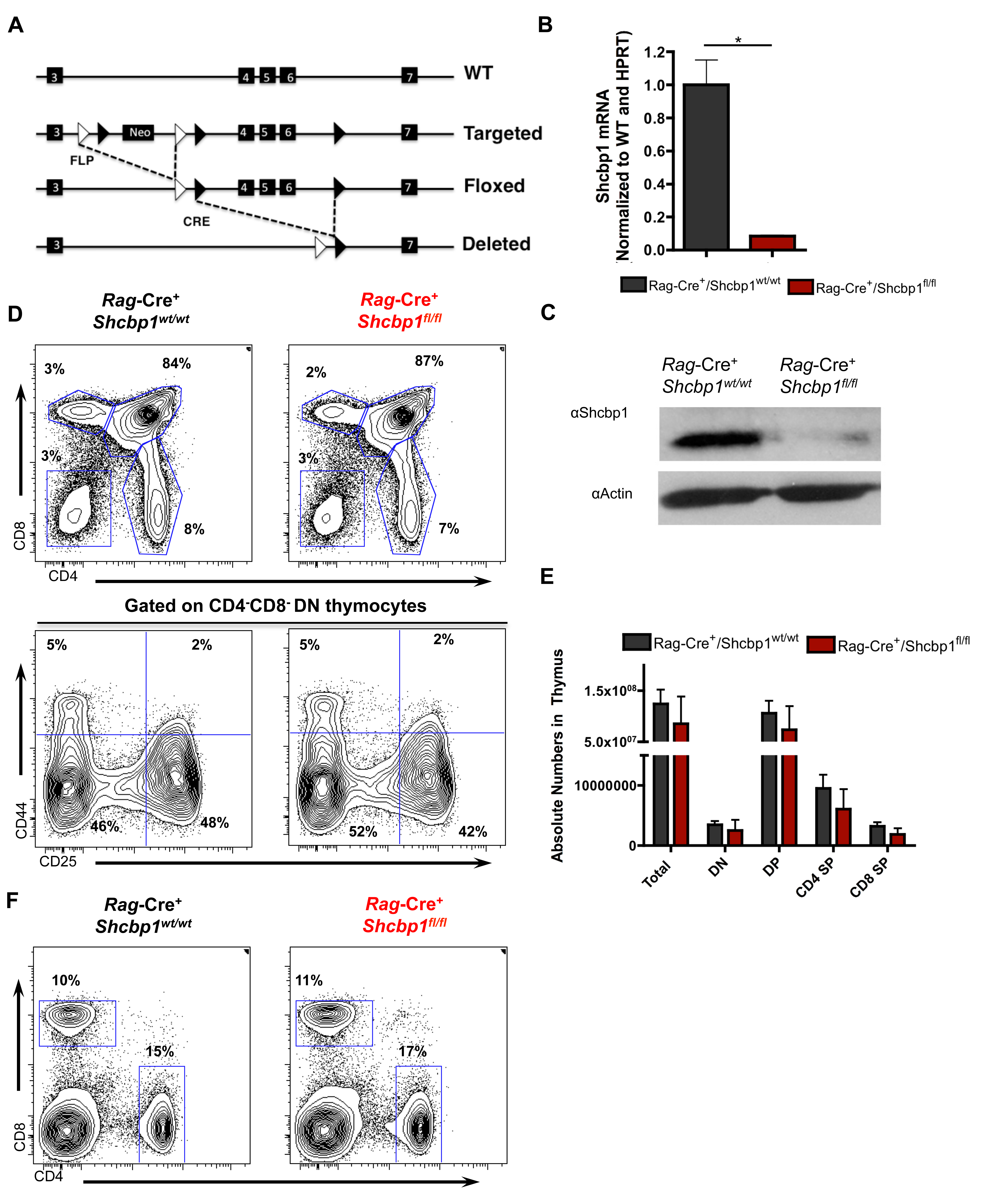

Supplement: Figure S4 — Generation and analysis of T cell development in the Rag-Cre+/Shcbp1fl/fl mice. (A) Strategy detailing the generation of mice with Shcbp1 conditionally deleted using the Rag-Cre. (B) Shcbp1 mRNA levels in thymocytes from Rag-Cre+/Shcbp1wt/w t and Rag-Cre+/Shcbp1fl/fl mice normalized to HPRT and to control (n>3 mice per genotype) (C) Immunoblotting of Shcbp1 in total thymocytes (n = 2 experiments). (D) Flow cytometric analysis of thymi isolated from 4-to-6 week old Rag-Cre+/Shcbp1wt/wt and Rag-Cre+/Shcbp1fl/fl mice. Top panel shows surface marker expression of CD4 and CD8. Bottom panel depicts surface marker expression of CD44 and CD25 gated on DN thymocytes (CD4− CD8− B220− Gr1− Ter119− CD11b− CD11c−) (n = 3–6 mice per genotype, age-matched littermate controls). (E) Total cellularity and absolute number of thymic subsets in 4-to 6-week-old Rag-Cre+/Shcbp1wt/wt and Rag-Cre+/Shcbp1fl/fl mice (n = 4–6 mice of each genotype with age-matched littermate controls). (F) Flow cytometric analysis for cell surface markers CD4 and CD8 in spleens isolated from 4–6 week old Rag-Cre+/Shcbp1wt/wt and Rag-Cre+/Shcbp1fl/fl mice (representative of n = 3–6 mice of each genotype, littermate controls). (TIF) [file pone.0105576.s004.tif]

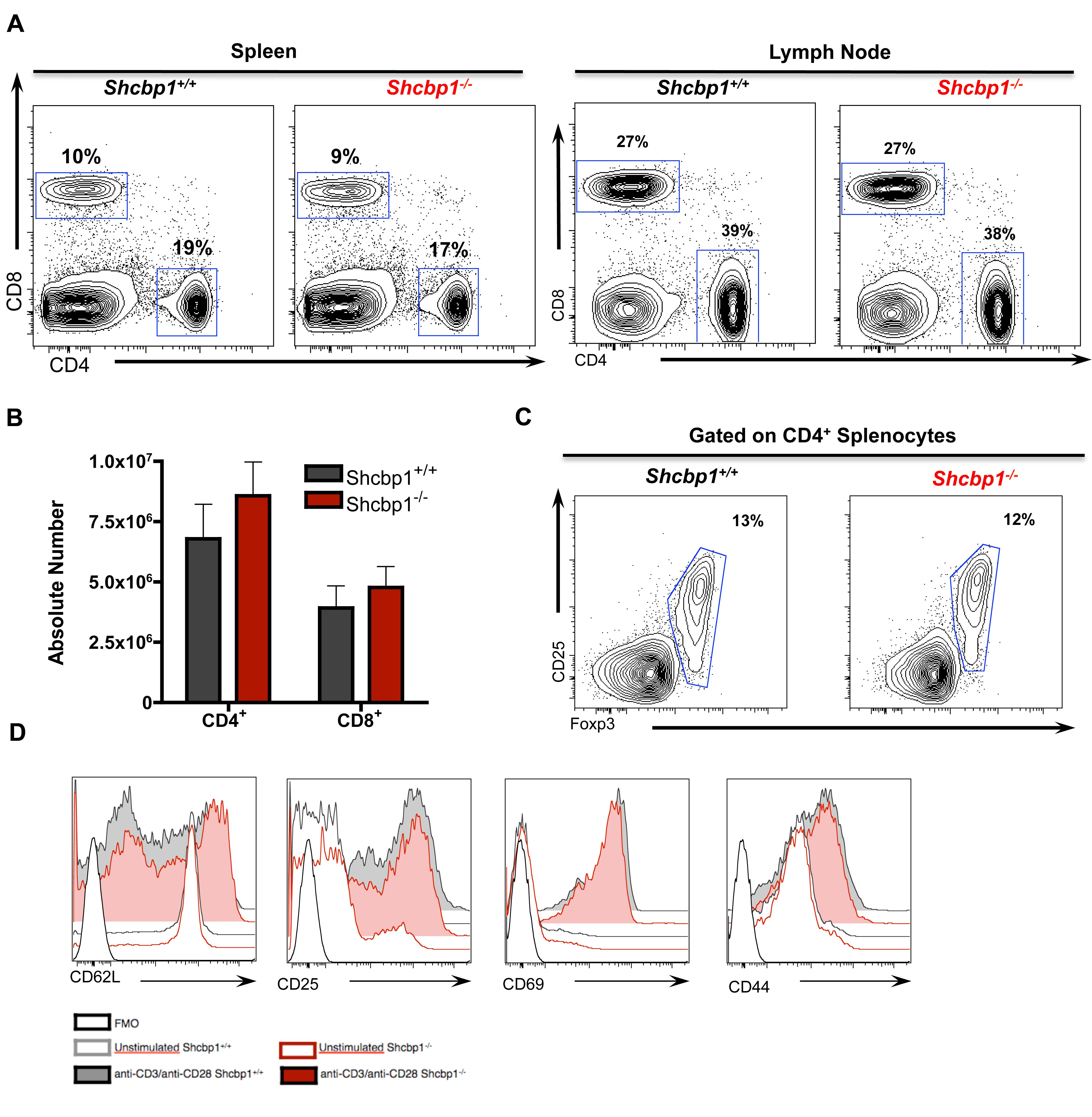

Supplement: Figure S5 — Peripheral compartment and activation of Shcbp1+/+ and Shcbp1−/− CD4+ T cells. (A) Surface staining, and (B) absolute numbers of CD4+ and CD8+ cells in spleen and lymph nodes of wild-type and Shcpb1 deficient mice (n>3 mice per genotype). (C) Intracellular staining for Foxp3 in CD4+ T cells from Shcbp1 WT and deficient mice (n = 2 mice per genotype). (D) Flow cytometry for cell surface markers (CD44, CD62L, CD25, and CD69, CD4) of CD4+ T cells isolated from Shcbp1+/+ and Shcbp1−/− mice after 24 hour stimulation with anti-CD3/anti-CD28 (n = 3 mice of each genotype). (TIF) [file pone.0105576.s005.tif]

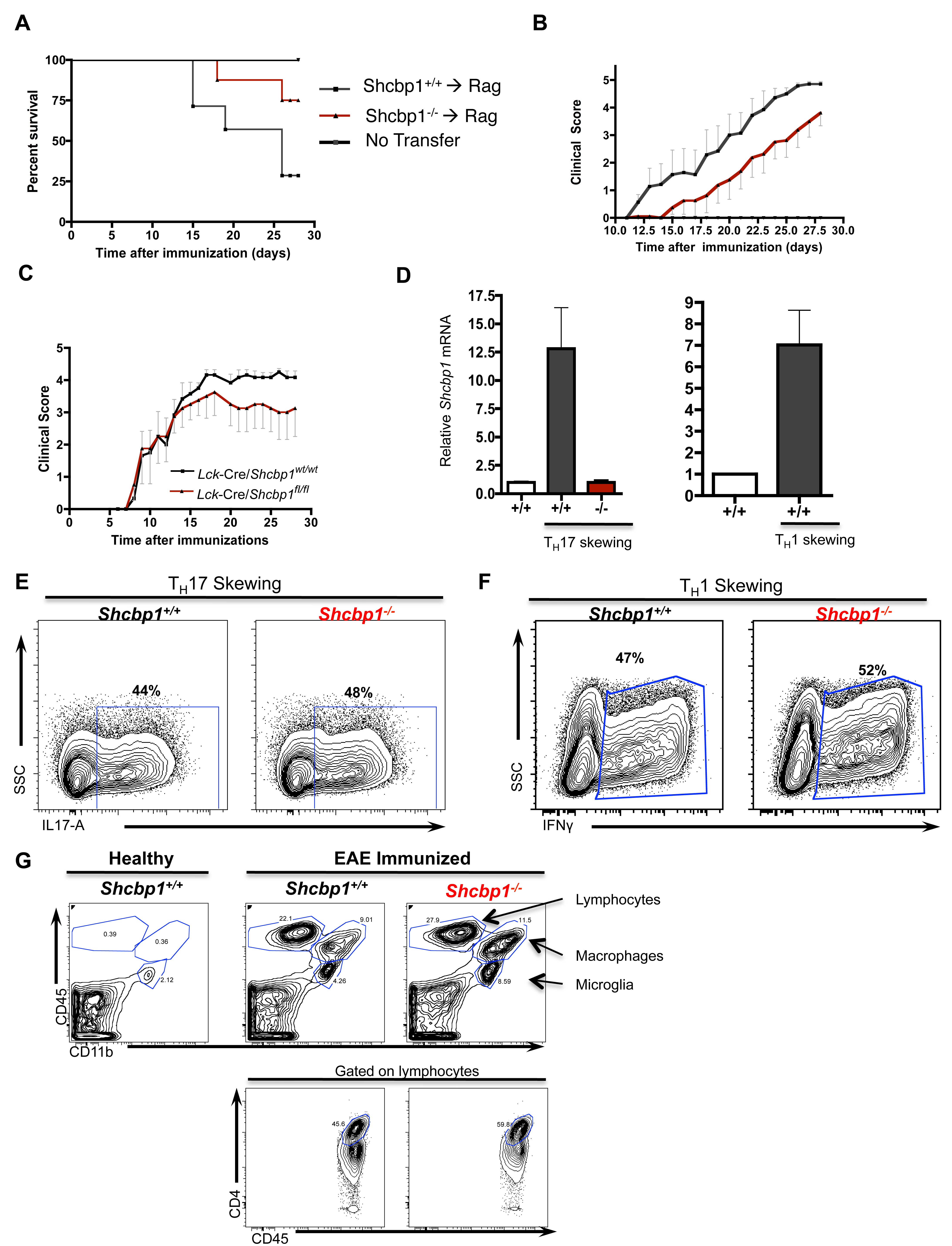

Supplement: Figure S6 — Shcbp1 expression specifically in T cells contributes to EAE disease severity. (A-B) Survival curves and clinical scores of Rag1 −/− mice after EAE induction one-week post transfer with CD4+ T cells isolated from either Shcbp1−/− or Shcbp1+/+ mice (n = 7, 8). (C) Clinical scores of Lck-Cre+/Shcbp1wt/wt and Lck-Cre+/Shcbp1fl/fl mice after EAE induction (n = 4,8). (D) RT-PCR for Shcbp1 in naïve or ex vivo TH17 or TH1 skewed CD4+ T cells (normalized to HPRT and unstimulated CD4+ T cells) (n = 2 mice of each genotype) (E-F) Intracellular staining for IL17-A or IFNγ in CD4+ T cells from Shcbp1+/+ and Shcbp1−/− mice after skewing (representative of n = 4 experiments with n = 4 mice of each genotype). (G) Cell surface staining for CD11b, CD45, and CD4 in mononuclear cells isolated from healthy controls or Shcbp1+/+ or Shcbp1−/− mice 28 days after EAE induction. (TIF) [file pone.0105576.s006.tif]

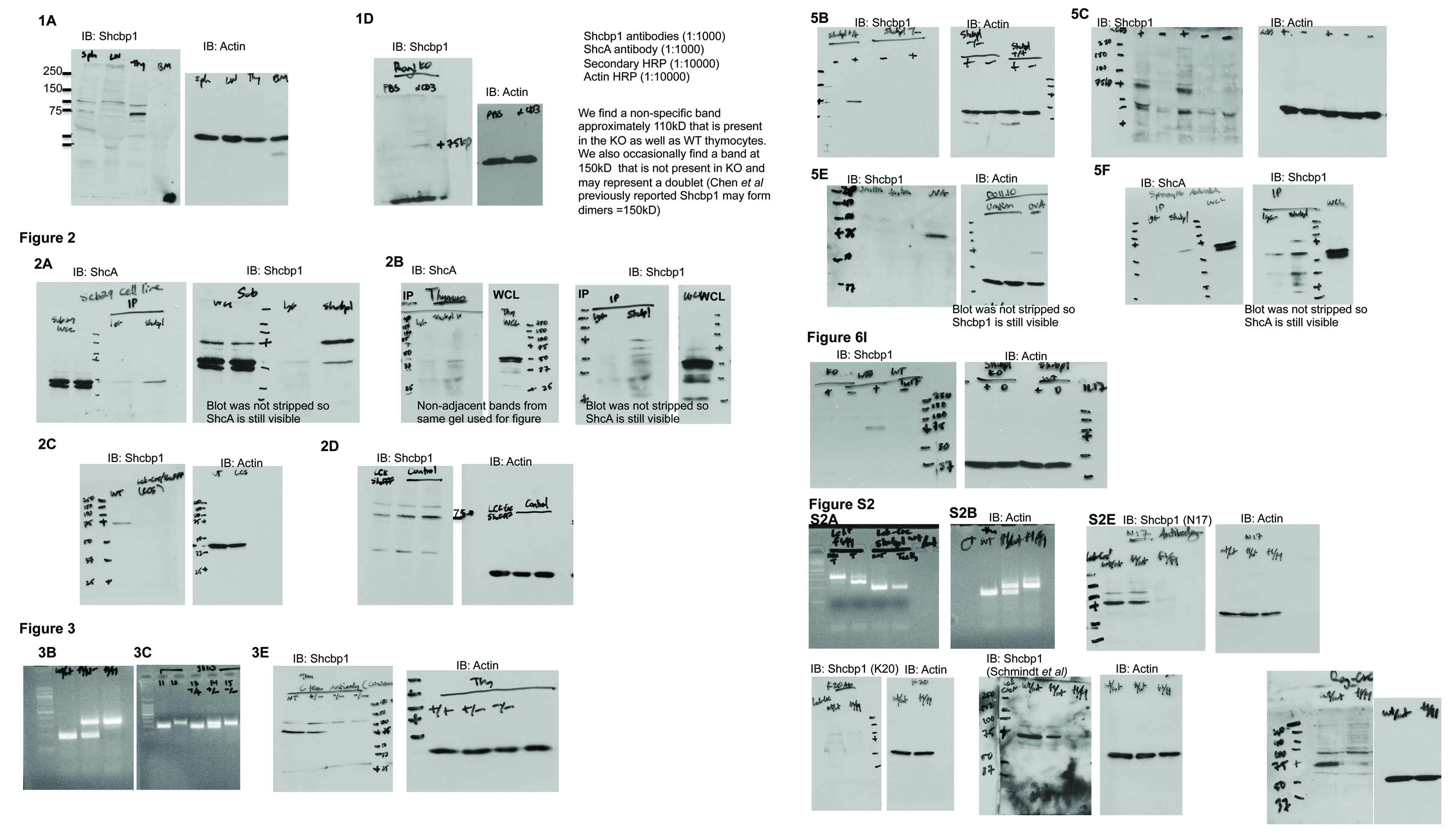

Supplement: Figure S7 — Original images and gels from all figures and supporting files. This supporting figure includes the original images and gels from all figures and supporting files. The images are not altered in any way and are unmodified and not cropped. (TIF) [file pone.0105576.s007.tif]
